# Physical, Volatile, and Flavour Precursor Changes in Faba Bean During *Rhizopus oryzae* Fermentation

**DOI:** 10.3390/foods15203683

**Published:** 2026-10-16

**Authors:** Jiaqiang Luo, Xueli Pang, Deepa Agarwal, Roman Buckow

**Affiliations:** 1 La Trobe Institute of Sustainable Agriculture and Food, La Trobe University, Bundoora, Melbourne, VIC 3083, Australia; pangxueli@caas.cn (X.P.); d.agarwal@latrobe.edu.au (D.A.); 2 Tobacco Research Institute, Chinese Academy of Agricultural Sciences, Qingdao 266101, China

**Keywords:** faba bean, fermentation, *Rhizopus oryzae*, volatile, metabolomics, texture, HS-SPME-GC–MS, LC–MS/MS

## Abstract

Faba bean (*Vicia faba* L.) is increasingly recognised as a nutritious and sustainable food ingredient, although its characteristic beany and green odours can limit consumer acceptance. Fermentation offers a potential strategy to modify the sensory and functional properties of legume-based foods. In this study, faba beans of different particle sizes, such as split beans (S), 4000–5000 μm (5K) and 1000–2000 μm (2K), were fermented with *Rhizopus oryzae* for up to 48 h to produce tempeh-like products. Mycelial development and colour were assessed by qualitative visual assessment and colour measurement, texture was evaluated at 40 and 48 h, volatile compounds were profiled using headspace solid-phase microextraction coupled with gas chromatography–mass spectrometry (HS-SPME-GC–MS), and free amino acids and fatty acids were quantified using liquid chromatography–tandem mass spectrometry (LC–MS/MS). Mycelial coverage increased throughout fermentation, with the S and 5K samples showing more extensive and uniform colonisation than 2K samples. Fermentation significantly altered colour, particularly through reduced yellowness, whereas texture showed no consistent differences between 40 and 48 h. Volatile profiles became increasingly diverse after 40 h, with fermentation time as the principal source of variation and particle size contributing to differences at 40 and 48 h. Changes in fatty acid and free amino acids were consistent with lipid transformation and amino acid catabolism. Several volatiles associated with beany and green odours increased rather than decreased during fermentation, providing no evidence for their direct removal. Overall, *R. oryzae* fermentation substantially altered the volatile profile of faba bean, suggesting that beaniness may be potentially masked by newly formed odourants rather than elimination of individual beany volatiles.

## 1. Introduction

The global shift towards plant-based diets has increased demand for diverse, sustainable and nutritionally valuable protein sources. Legumes have long formed an important component of plant-based nutrition, with soybean remaining one of the dominant protein crops because of its long history of cultivation and significant economic importance. However, concerns regarding the environmental impacts associated with intensive soybean production, including deforestation, biodiversity loss, and resource use, have encouraged greater interest in alternative legumes that combine nutritional quality with ecological benefits [1]. Among these, faba beans (*Vicia faba* L.) have gained increasing attention because of their nutritional, functional and agronomic attributes. This growing interest has been accompanied by increased production in major legume-producing countries, including Canada and Australia [2,3].

Despite these advantages, the characteristic off odours and flavours of faba beans remain a major barrier to consumer acceptance and broader food application. These undesirable sensory attributes are commonly described as “beany,” “green,” “grassy,” or “rancid” and are largely associated with lipid oxidation and the degradation of amino acids and unsaturated fatty acids during seed development, storage, and processing [4]. Important volatile contributors include aliphatic aldehydes such as hexanal and nonanal, together with alcohols, ketones, and furans generated through oxidation and related metabolic pathways [4]. Although faba beans contain relatively low total lipid levels compared with oil-rich legumes, their lipids are rich in mono- and polyunsaturated fatty acids, which account for more than 80% of total fatty acids and are particularly susceptible to oxidation [5]. These unsaturated lipids therefore represent important precursors for the formation of odour-active compounds.

A range of processing, formulation and genetic strategies has been investigated to mitigate undesirable flavour in faba bean ingredients. Thermal treatments, including roasting and heat processing of protein-rich fractions, can reduce undesirable sensory attributes through the inactivation of lipid-degrading enzymes and modification of volatile compounds associated with green and rancid notes [6]. Processing variables such as pH, temperature and treatment duration can similarly alter the formation, stability and release of odour-active molecules, thereby influencing the overall aroma profile [7]. Fermentation represents another promising strategy because microbial metabolism can transform existing volatile compounds and generate new aroma-active metabolites, potentially reducing the perceived intensity of characteristic beany notes [8]. In parallel, breeding approaches are being explored to improve sensory quality at the crop level. Lippolis et al. [9] demonstrated the potential to reduce undesirable aroma characteristics by targeting metabolic pathways associated with lipid oxidation. Subsequent work identified genetic markers linked to flavour-related traits that may facilitate the development of cultivars with improved sensory characteristics [10].

Among fermentation-based approaches, tempeh provides a useful model for developing structured legume-based foods. Traditional tempeh is produced through solid-state fermentation of boiled soybeans with *Rhizopus* spp., during which fungal hyphae proliferate across and between the substrate particles to form a cohesive tempeh-like structure. During this process, extracellular proteases, lipases, and other hydrolytic enzymes modify protein, lipid and carbohydrate fractions, thereby altering the nutritional, structural and aroma characteristics of the substrate. Previous studies of soybean and barley tempeh have demonstrated that fermentation time and substrate composition influence the formation of alcohols, ketones, esters, aldehydes, and other volatile compounds [11,12].

Faba bean is also a suitable substrate for tempeh-like fermentation. Fermentation with different strains of *R. oligosporus* has been reported to modify fatty acid, amino acid, phenolic and sterol profiles, demonstrating the potential of faba bean as an alternative substrate for tempeh production [13]. More recently, solid-state fermentation of faba bean flour with *R. oligosporus* or *Aspergillus oryzae* was shown to alter nutritional, functional and volatile characteristics [14]. However, comparatively little is known about how fungal colonisation and tempeh-like structure development relate to changes in volatile composition and to the corresponding amino acid and fatty acid precursor pools during fermentation.

Particle size may be particularly important in this context because it influences the physical environment of solid-state fermentation. Changes in particle size affect surface area, particle packing, bed porosity, moisture distribution, oxygen transfer and nutrient accessibility. Although smaller particles provide greater surface area for fungal attachment and enzymatic interaction, excessive size reduction can increase packing density and restrict gas transfer. In contrast, larger particles may provide improved porosity and aeration but reduce the available surface area for colonisation. Particle size has previously been shown to affect fungal biomass production in solid-state fermentation with *R. oryzae*, highlighting the importance of substrate structure in determining fermentation performance [15,16]. Nevertheless, its influence on the physical development, texture and volatile profile of faba bean tempeh-like products has not been systematically investigated.

Building on our previous work examining the effects of particle size and *Rhizopus* species on fermentability, mycelial development, protein structure, antioxidant activity, antinutritional factors, and rheological properties of fermented faba bean [16], the present study further extends this system to the development of physical and volatile characteristics. Faba bean substrates of different particle sizes were fermented with *R. oryzae* for up to 48 h, and changes in visual appearance, colour and texture were evaluated. Volatile compounds were semi-quantified using gas chromatography–mass spectrometry (HS-SPME-GC–MS), while selected free amino acids and fatty acids were quantitatively determined using liquid chromatography–tandem mass spectrometry (LC–MS/MS). By integrating physical, volatile and precursor measurements, this study aims to determine how fermentation time and particle size influence the development of faba bean tempeh-like products and to characterise changes in the volatile profile and selected flavour precursors during fermentation.

## 2. Materials and Methods

### 2.1. Chemicals

Analytical-grade sodium chloride, the internal standard nitrobenzene, amino acid standards, a C7–C40 mixed alkane standard, ammonium acetate, N-(3-Dimethylaminopropyl)-N′-ethylcarbodiimide (EDC) and pyridine were purchased from Merck (St. Louis, MO, USA). All 50 fatty acid standards and 3-Nitrophenylhydrazine-d4 (3-NPH-d4) were obtained from Zhenzhun Standards Co., Ltd. (Shanghai, China). Isopropanol (Optima LC-MS), methanol (Optima LC-MS), acetonitrile (Optima LC-MS), and formic acid (Optima LC-MS) were purchased from Thermo-Fisher Scientific (FairLawn, NJ, USA). Purified water was obtained from a Milli-Q system (Millipore Australia, Bayswater, VIC, Australia).

### 2.2. Faba Beans and Starter Culture

Split faba beans were purchased from Essantis (Melbourne, VIC, Australia). A commercial *R. oryzae* starter culture was obtained from Naturita (Amsterdam, The Netherlands).

### 2.3. Preparation of Faba Bean Substrates with Different Particle Sizes

The preparation procedure generally followed our previous work [16] and was conducted in four replicates. Briefly, three faba bean particle size fractions were prepared: split beans (S), 4000–5000 μm (5K), and 1000–2000 μm (2K). The 5K and 2K fractions were obtained using a sieve set comprising 1000, 2000, 4000, and 5000 μm mesh sizes. Coarsely ground split faba beans were loaded onto the sieves and separated by continuous manual shaking. Fifty grams of each faba bean fraction were evenly distributed into polypropylene containers (144 mm × 60 mm × 105 mm) and sterilised by dry autoclaving at 121 °C for 15 min. After cooling to room temperature, autoclaved Milli-Q water was added to each sample. Water volumes and soaking times were adjusted according to particle size as shown in Table 1. These conditions were selected to achieve an approximate moisture content of 50%. Although the water-to-bean ratio and soaking time differed between 2K and the other two treatment groups, these adjustments were necessary to obtain comparable starting moisture contents before fermentation.

### 2.4. Inoculation and Fermentation

A solid-state fermentation approach was employed. *R. oryzae* (1.16 × 10^9^ spores/mL) was inoculated into each sample at a culture-to-substrate ratio of 1:200. Following inoculation, each container was covered with perforated aluminium foil to allow passive aeration during solid-state fermentation. Samples were incubated in a static incubator (Thermo Fisher Scientific, Waltham, MA, USA) at 25 °C for 0, 24, 40, and 48 h. In our preliminary study, fermentation beyond 48 h resulted in fungi sporulation, making the sample unsuitable as a food product model due to discolouration. In addition, commercial tempeh fermentation is commonly controlled within 48 h. Therefore, 48 h was selected as the maximum fermentation time. At each designated time point, samples intended for colour and texture analyses were immediately removed from the incubator and stored at 4 °C until analysis. Samples designated for HS-SPME-GC–MS and LC–MS/MS analyses were stored at −20 °C until analysis. This experimental design resulted in a total of 12 treatment groups as provided in Table 1.

### 2.5. Visual Inspection and Colour Analysis

Samples were equilibrated to room temperature before analysis. Mycelial development was visually assessed and photos were taken from the top and side views. For the 0 h and 24 h treatment groups, each sample was loaded into a transparent Petri dish (5 cm i.d.) until the bottom of the dish was completely covered. For the 40 h and 48 h treatment groups, the 3 cm-diameter sample tempeh was pressed firmly into the glass Petri dish. Petri dishes containing samples were then placed over the sensor port of a ColorFlex L2 colourimeter (HunterLab, Reston, VA, USA). Measurements were performed in triplicate. Results were expressed as International Commission on Illumination (CIE) L*, a*, and b* scales. Colour analysis was performed on all four biological replicates for each treatment were subjected to colour analysis.

### 2.6. Texture Analysis

Samples were equilibrated to room temperature before analysis. Texture analysis was performed using a TA.XTplusC Texture Analyser (Stable Micro Systems, Godalming, UK). Measurements were conducted only on samples fermented for 40 and 48 h, as earlier fermentation stages had not developed a sufficiently cohesive tempeh structure for reliable testing. Prior to analysis, samples were cut into circular cakes (3 cm diameter) using a cylindrical cutter. Three cakes were collected from the top left, centre and bottom right regions of each fermented cake and analysed as analytical replicates. Texture profile analysis was performed using a double compression test with an SMS P/35 cylindrical probe attached to a 10 kg load cell. Samples were compressed to 40% strain, and hardness, adhesiveness, resilience, cohesiveness, springiness, gumminess and chewiness were calculated using the instrument software and built-in texture profile analysis macro. All four biological replicates were analysed for each treatment.

### 2.7. Volatile Profiling by HS-SPME-GC–MS

Frozen samples were ground into a fine powder using a grinder. For HS volatile analysis, 2 g of powdered sample was transferred into a 20 mL headspace vial and mixed with 3 mL of saturated NaCl solution to limit enzymatic and microbial activity [17]. In preliminary experiments, 10 μL of nitrobenzene solution (20 mg/L) was added as an internal standard. However, a pronounced matrix effect on nitrobenzene partitioning was observed, and therefore no internal standard was used in the final analysis. Samples were incubated at 40 °C for 15 min with continuous agitation at 350 rpm. Subsequently, a 1.10 mm DVB/C-WR/PDMS SPME Arrow fibre (120 μm × 20 mm; Thermo Fisher Scientific) was exposed to the headspace of the vial for 40 min at 40 °C. Extraction was performed using a TriPlus RHS multipurpose autosampler coupled to a TRACE 1300 Series gas chromatograph and a TSQ 8000 Series triple quadrupole mass spectrometer (Thermo Fisher Scientific).

The SPME fibre was desorbed in the GC inlet at 260 °C for 10 min. Volatile compounds were separated using a semi-polar Agilent J&W VF-5ms column (30 m × 0.25 mm × 0.25 μm), equipped with a 10 m EZ-Guard column. Ultra-high purity helium (Coregas, Thomastown, Australia) was used as the carrier gas at a constant flow rate of 1.0 mL/min. The GC oven temperature programme started at 35 °C and was held for 6 min, followed by an increase to 150 °C at a rate of 5 °C/min and a hold of 2 min. The oven temperature was then ramped to 320 °C at 20 °C/min and held for 3 min. Mass spectra were acquired using electron impact ionisation at 70 eV over an m/z range of 35–450, with a solvent delay of 2 min. The transfer line, ion source, and quadrupole temperatures were set at 320 °C, 250 °C, and 248 °C, respectively.

A C_7_–C_40_ mixed alkane standard was analysed to determine the retention indices (RIs) of volatile compounds. Tentative compound identification was performed by comparing electron impact mass spectra and RI data with records in the National Institute of Standards and Technology (NIST) library, version 20. Peak integration was carried out using the responses of selected target ions. Semi-quantification was based on the mean peak responses of individual volatile compounds across four replicates. Detailed information on compound identification is provided in Supplementary Table S1.

### 2.8. Quantification of Free Amino Acids and Free Fatty Acids by LC–MS/MS

Samples were freeze-dried prior to analysis. For free amino acid analysis, 20 mg of each freeze-dried sample was accurately weighed, ground under liquid nitrogen, and resuspended in water by thorough vortexing to obtain diluted sample extracts (1:50, sample:water, *w*/*w*). An aliquot of 50 μL of each diluted extract was mixed with 200 μL of acetonitrile/methanol (1:1, *v*/*v*). The mixture was kept on ice for 30 min, followed by centrifugation at 12,000 rpm for 10 min. The supernatant was then collected and injected into the LC–MS/MS system for analysis.

For free fatty acid analysis, 20 mg of each freeze-dried sample was ground under liquid nitrogen and homogenised with isopropanol/acetonitrile (1:1, *v*/*v*), followed by ultrasonic treatment for 10 min. The mixture was kept at −20 °C for 60 min and then centrifuged at 12,000 rpm for 15 min. An aliquot of 50 μL of the supernatant was mixed with 150 μL of derivatisation reagent consisting of 50 μL of 160 mM 3-NPH-d4 in 80% methanol, 50 μL of 120 mM EDC in methanol and 50 μL of 8% pyridine in methanol and derivatised at 40 °C for 40 min. The derivatised sample was diluted with isopropanol/acetonitrile (1:1, *v*/*v*), after which 95 μL of the supernatant was mixed with 5 μL of mixed internal standard solution and injected into the LC–MS/MS system for analysis.

Both analyses were conducted using ultra-high-performance liquid chromatography coupled with tandem mass spectrometry (ExionLC AD UHPLC-QTRAP 6500+, AB SCIEX Corp., Boston, MA, USA). For free amino acids, chromatographic separation was performed on an ACQUITY UPLC BEH Amide column (2.1 × 100 mm, 1.7 μm) maintained at 50 °C. The mobile phase consisted of 0.1% formic acid in 5 mM ammonium acetate (solvent A) and 0.1% formic acid in acetonitrile (solvent B), delivered at a flow rate of 0.30 mL/min. The solvent gradient started at 80% solvent B, which was held for 0.5 min, followed by a decrease to 70% over 2 min and then to 45% over the subsequent 4 min. The proportion of solvent B was then increased back to 80% and maintained until 9 min. The mass spectrometer was operated in positive multiple reaction monitoring mode, with an ion spray voltage of 5500 V, curtain gas pressure of 35 psi, ion source temperature of 550 °C, and ion source gas 1 and gas 2 pressures of 50 and 60 psi, respectively.

For free fatty acids, chromatographic separation was performed on a Waters ACQUITY UPLC BEH C18 column (2.1 × 100 mm, 1.7 μm) maintained at 40 °C. The mobile phase consisted of 0.1% formic acid in acetonitrile/water (1:1, *v*/*v*; solvent A) and isopropanol/acetonitrile (1:1, *v*/*v*; solvent B), delivered at a flow rate of 0.30 mL/min. The solvent gradient began at 45% solvent B, which was held for 1 min, followed by increases to 70% at 4.5 min, 75% at 9 min, 80% at 12.5 min, and 100% at 14 min. The proportion of solvent B was then reduced to 45% at 15.1 min and maintained until 17 min. The mass spectrometer was operated in negative multiple reaction monitoring mode, with an ion spray voltage of −4500 V, curtain gas pressure of 35 psi, ion source temperature of 550 °C, and ion source gas 1 and gas 2 pressures of 60 psi.

Both analyses were performed by Novogene Co., Ltd. (Beijing, China) on three biological replicates. Detailed concentration data for all analysed samples expressed on a fresh weight basis, together with the corresponding moisture contents are provided in Supplementary Table S2. Fresh weight basis concentrations were calculated using the following equation:

$${\mathrm{Concentration}}_{\mathrm{fresh}\;\mathrm{basis}}={\mathrm{Concentration}}_{\mathrm{dry}\;\mathrm{basis}}\;\times \;\frac{100-\mathrm{moisture}\;\left(\%\right)}{100}$$

### 2.9. Statistical Analyses

For detailed comparisons among colour, texture, volatile compounds and precursor metabolites, treatment effects were evaluated using one-way analysis of variance (ANOVA), followed by Fisher’s least significant difference (LSD) post hoc test as an exploratory statistical analysis. The effects of particle size, fermentation time and their interaction on individual colour and texture parameters, as well as volatiles, were further assessed using factorial permutation F-tests on log1p-transformed data. The resulting *p*-values were adjusted across compounds using the Benjamini–Hochberg false discovery rate (FDR) procedure. Principal component analysis (PCA) was performed on z-standardised volatile data to visualise similarities and differences among samples and identify compounds contributing to sample separation. The FDR-significant volatiles specific to the particle size × fermentation time treatment group, identified using permutation-based group versus rest contrasts with global Benjamini–Hochberg correction (*q* < 0.05), were displayed as loading vectors in the PCA biplot. Precursors associated with the selected volatiles were identified from their annotated metabolic pathways and presented in a heatmap. All statistical analyses and visualisations were performed using Python version 3.12.13 (Python Software Foundation, Beaverton, OR, USA).

## 3. Results and Discussion

### 3.1. Mycelial Development

**Figure 1:**
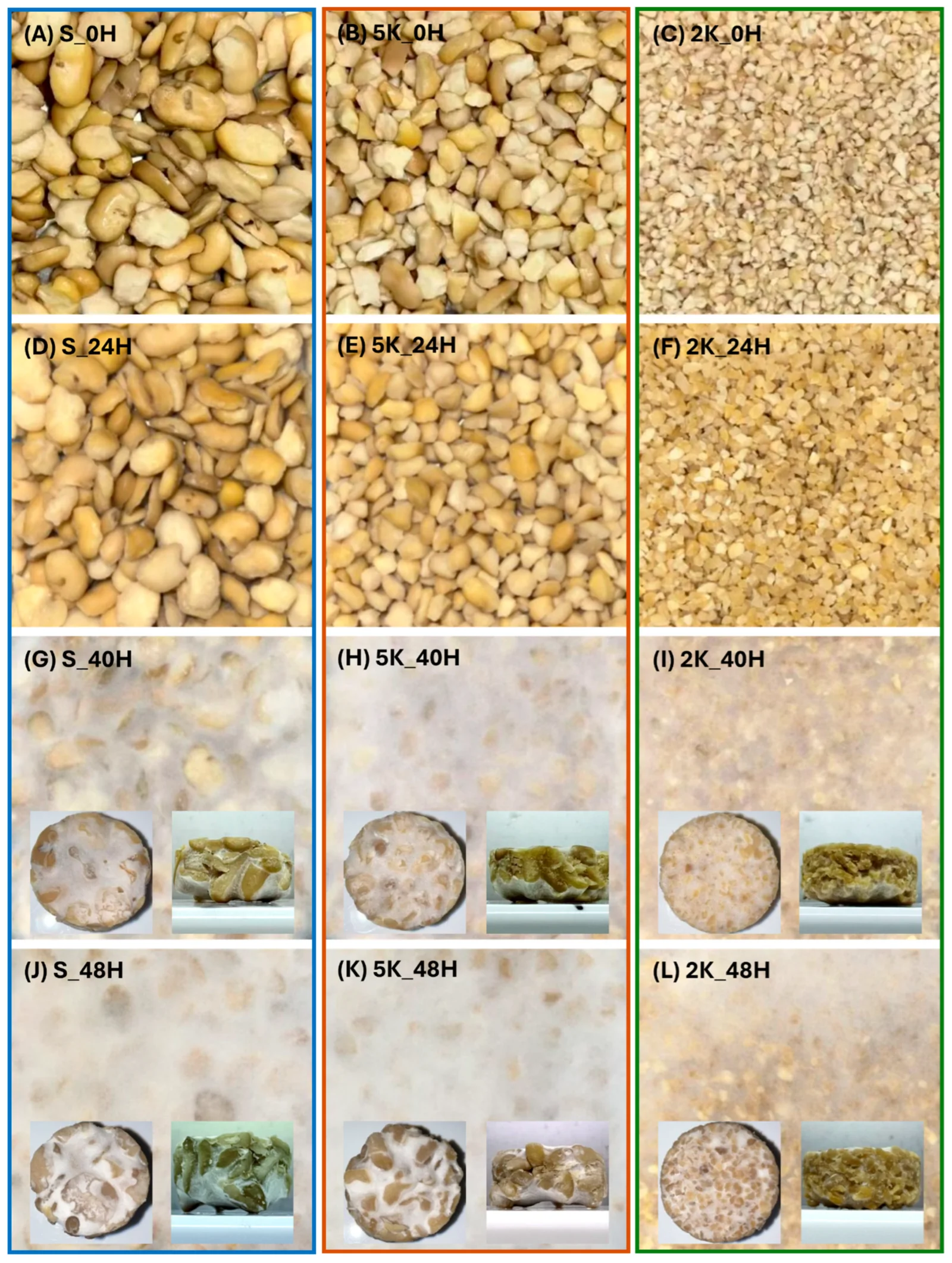
Top views of faba bean samples with different particle sizes (S, split beans; 5K, 4000–5000 μm; and 2K, 1000–2000 μm) during fermentation. (**A**) S_0H, (**B**) 5K_0H, (**C**) 2K_0H, (**D**) S_24H, (**E**) 5K_24H, (**F**) 2K_24H, (**G**) S_40H, (**H**) 5K_40H, (**I**) 2K_40H, (**J**) S_48H, (**K**) 5K_48H, and (**L**) 2K_48H. Additional top and side views of 3 cm-diameter cakes from the 40 and 48 h treatments are shown as insets in the corresponding panels. Figures representing S, 5K and 2K treatment groups are highlighted in blue, red and green, respectively.

Representative images of faba bean samples with different particle sizes during fermentation are shown in Figure 1. At 0 h (Figure 1A–C), the split bean (S) samples comprised the largest and most intact cotyledon pieces, whereas the 5K and 2K treatments contained progressively smaller particles and appeared more densely packed. After 24 h (Figure 1D–F), all treatments remained visually similar to their respective unfermented samples, with only limited visible mycelial development. By 40 h (Figure 1G–I), fungal growth was clear across all treatments. The S and 5K samples exhibited extensive white mycelial coverage, with hyphae bridging adjacent particles and forming cohesive tempeh-like structures. In contrast, colonisation of the 2K samples was less uniform, with individual fine particles remaining distinguishable. After 48 h (Figure 1J–L), mycelial coverage increased further, particularly in the S and 5K treatments, where dense white mycelia extended across much of the surface and cross section. The 2K samples remained comparatively less densely and continuously colonised. Overall, mycelial development increased with fermentation time, while particle size influenced the extent and uniformity of visible fungal colonisation. The progressive development of mycelia is consistent with the characteristic growth of *Rhizopus* spp. during tempeh fermentation, in which fungal hyphae extend through the substrate and progressively bind individual particles into a continuous matrix [14,18]. The greater visible colonisation of the S and 5K treatments may be associated with differences in substrate packing and aeration. Coarser particles are likely to retain larger interparticle voids, thereby facilitating oxygen transfer and hyphal extension, whereas finer particles may pack densely and restrict gas movement through the substrate. Oxygen limitation has previously been reported within dense and moist fungal layers during solid-state fermentation [19], while particle size has also been shown to influence biomass production by *R. oryzae* [15]. The present observations therefore suggest that substrate particle size influences mycelian development, potentially through its effects on bed porosity and oxygen availability. This interpretation is consistent with our previous study, in which coarser faba bean particles supported more uniform mycelial network formation, particularly during fermentation with *R. oryzae* [16].

### 3.2. Colour Changes

Colour parameters of faba bean samples were significantly affected by fermentation time, particle size and their interaction (*q* < 0.05; *p* < 0.05) (Table 2). In the CIE Lab* colour space, L* represents lightness (0 = black, 100 = white), a* represents the green to red axis (negative = green, positive = red), and b* represents the blue to yellow axis (negative = blue, positive = yellow). At 0 h, the 2K treatment showed the highest L* value, followed by 5K and S, indicating that the smaller substrate initially had a lighter appearance. Changes in L* during fermentation were dependent on particle size. Lightness of the S samples remained comparatively stable, ranging from 56.05 to 58.09 throughout fermentation. In the 5K samples, L* decreased slightly at 24 h before increasing at 40 and 48 h, reaching 61.37 at 48 h. In contrast, L* progressively decreased in the 2K samples, from 64.70 at 0 h to 56.53 at 48 h.

The a* values generally decreased during fermentation in the S and 5K treatments. For S, a* decreased from 5.92 at 0 h to 4.14 at 48 h, while the corresponding decrease in 5K was from 5.08 to 3.49. In contrast, the 2K treatment showed a less consistent response, with a* increasing at 24 h, decreasing at 40 h and increasing again at 48 h. The clearest fermentation-related change was observed for b*. Values decreased markedly across all particle size treatments, from 31.99 to 19.16 for S, 31.43 to 18.74 for 5K and 31.80 to 20.79 for 2K between 0 and 48 h. These results indicate a substantial loss of yellowness during fermentation, particularly during the later stages.

Among the measured colour parameters, b* therefore showed the most consistent response to fermentation, whereas changes in L* and a* were more dependent on particle size and fermentation stage. Fermentation-induced colour changes have also been reported for other tempeh systems. Handoyo and Morita [20] reported that soybean tempeh became darker during fermentation, as reflected by decreasing L* and increasing a* values. They associated this change with fungal maturation, particularly the increasing proportion of mature, darker *R. oligosporus* relative to white immature mycelia. In fermented faba bean flour, Gautheron, Nyhan [14] reported decreased L* and increased a* and b* following fermentation with *R. oligosporus,* with the changes attributed to fungal mycelia and spore development. The differences between these studies and the present findings may reflect differences in fungal species, fermentation substrate, particle size, fermentation conditions, and sample presentation. In the present system, the marked decrease in b* suggests that fermentation substantially modified the native yellow appearance of the faba bean substrate, while the variable L* response indicates that lightness was influenced by both fungal development and the physical characteristics of the substrate.

### 3.3. Texture Profile Analysis

Texture analysis was restricted to samples fermented for 40 and 48 h because earlier samples had not developed a sufficiently cohesive structure for reproducible testing (Table 2). At 40 h, hardness ranged from 3318.82 to 3691.88 g, increasing numerically to 3683.80 to 4378.28 g at 48 h. Despite this increase across all particle size treatments, no significant differences in hardness were detected among samples (*p* > 0.05). Gumminess and chewiness similarly increased numerically in the S and 5K treatments after 48 h, although these differences were not statistically significant. Significant differences (*p* < 0.05) were observed among treatments for adhesiveness, resilience, cohesiveness, and springiness, while particle size had significant effects (*q* < 0.05) on adhesiveness, resilience, and cohesiveness. Adhesiveness ranged from −9.37 to −19.11 g·s at 40 h and from −11.85 to −18.10 g·s at 48 h. The 5K_48H and 2K_40H samples exhibited the greatest magnitude of adhesiveness, whereas 5K_40H showed the lowest. Resilience and cohesiveness were highest in the 5K_48H and 2K_40H samples. Springiness ranged from 49.55% to 58.22%, with the highest values observed for 2K_40H and S_48H and the lowest for 2K_48H. However, these differences did not follow a consistent pattern with either fermentation time or particle size.

The development of a measurable tempeh structure by 40 h coincided with the appearance of an extensive visible mycelial network. During tempeh fermentation, fungal hyphae grow between substrate particles and bind them into a continuous matrix that contributes to product cohesion and mechanical integrity [14,20]. The absence of significant differences in hardness, gumminess and chewiness between 40 and 48 h suggests that much of the structural development required to produce a cohesive tempeh had already occurred by 40 h. The subsequent 8 h of fermentation therefore produced only limited additional changes in bulk texture, despite continued visible mycelial growth. The significant but non-systematic differences in adhesiveness, resilience, cohesiveness and springiness further suggest that local variations in particle arrangement and mycelial distribution may contribute to textural heterogeneity within the fermented matrix.

These findings complement our previous rheological study, in which particle size and *Rhizopus* species interacted to influence the yield stress of fermented faba bean, with samples fermented by *R. oligosporus* generally exhibiting higher yield stress than those fermented by *R. oryzae* [16]. Although yield stress and texture profile parameters describe different aspects of material behaviour and should not be directly equated, the combined findings indicate that the substrate structure and fungal species both contribute to the mechanical properties of fermented faba bean matrices.

### 3.4. Changes in Volatile Profile of Faba Bean Tempeh During Fermentation

A total of 1 acid, 11 alcohols, 14 aldehydes, 12 ketones, 2 esters, 2 furans, 2 S-containing compounds, 3 N-containing compounds, 3 terpenes, and 1 hydrocarbon were detected in the HS-SPME-GC–MS analysis (Table 3). Overall, fermentation resulted in a more diverse volatile profile, with most significant changes (*p* < 0.05) occurring between 24 and 40 h. Notably, fermentation time alone had significant effects (*q* < 0.05) on 46 of the 51 detected compounds, followed by the fermentation time × particle size interaction, which affected 20 compounds. In contrast, particle size alone significantly affected only α-pinene and pentanal.

Only one acid, 3-methylbutanoic acid, was detected. Its abundance was highest in the early stage samples, particularly 2K_0H and S_24H, and generally decreased during fermentation. It was not detected in the 2K samples after 24 h. In contrast, most alcohols increased during fermentation. 3-Methyl-1-butanol, 2-methyl-1-butanol, 1-pentanol, 1-hexanol, 3-octanol, 2-ethyl-1-hexanol, and 1-octanol increased substantially after 24 h. The unsaturated alcohol (*E*)-2-octen-1-ol was not detected at 0 or 24 h but appeared at 40 h. 1-Octen-3-ol showed one of the largest increases in the peak response in the S_40H and S_48H samples and similarly high values in the 5K samples. Its abundance was lower in the 2K treatment and decreased between 40 and 48 h. 2-Butanol also increased markedly and reached its highest value in the 2K_48H sample.

Most aldehydes increased during the later stages of fermentation. The branched-chain aldehydes 3-methylbutanal and 2-methylbutanal increased sharply from 0 to 40 h and then decreased at 48 h, while remaining higher than in the unfermented samples. Hexanal, heptanal, octanal, nonanal, and decanal generally increased after 24 h, particularly in the S and 5K treatments. Several unsaturated aldehydes, including (*E*)-2-octenal, (*E*)-2-nonenal, and (*Z*)-4-decenal, appeared or increased substantially after 40 h. Pentanal and (*Z*)-2-heptenal showed more variable treatment-dependent patterns.

The ketone compounds showed both increasing and decreasing trends. 2,3-Butanedione generally decreased during fermentation, whereas 2-butanone increased markedly from 24 h onwards. 3-Pentanone increased sharply and reached its highest abundance at 40 h before declining at 48 h. Fungal-associated ketones, including 1-octen-3-one, 3-octanone, 2,3-octanedione, 3-octen-2-one, and 6-methyl-5-hepten-2-one, appeared or increased mainly after 24 h. These compounds generally showed higher abundances in the S and 5K treatments than in the 2K treatment at 48 h.

The ester and furan compounds also generally increased during fermentation. Methyl 3-methylbutanoate increased substantially during fermentation and reached high values at 40 h, particularly in the S and 5K treatments, before declining at 48 h. Methyl heptanoate was not detected in the unfermented samples but increased at 40 and 48 h. Similarly, 2-butylfuran and 2-pentylfuran generally increased during fermentation, with the highest values mostly observed in the S treatment.

Methional was not detected at 0 or 24 h but appeared after 40 h. Dimethyl disulphide showed considerable numerical variation but no significant differences among treatments. The N-containing compounds generally increased after 24 h. Methylpyrazine, 2,6-dimethylpyrazine, and 2,3,5-trimethylpyrazine were generally most abundant in the S and 5K treatments, although some compounds reached their maximum at 40 h and declined slightly at 48 h. The terpene compounds *β*-ocimene and D-limonene showed no clear fermentation-related trend, while α-pinene tended to decrease in the 2K samples. Ethylbenzene generally increased during fermentation, particularly in the S and 5K treatments.

**Figure 2:**
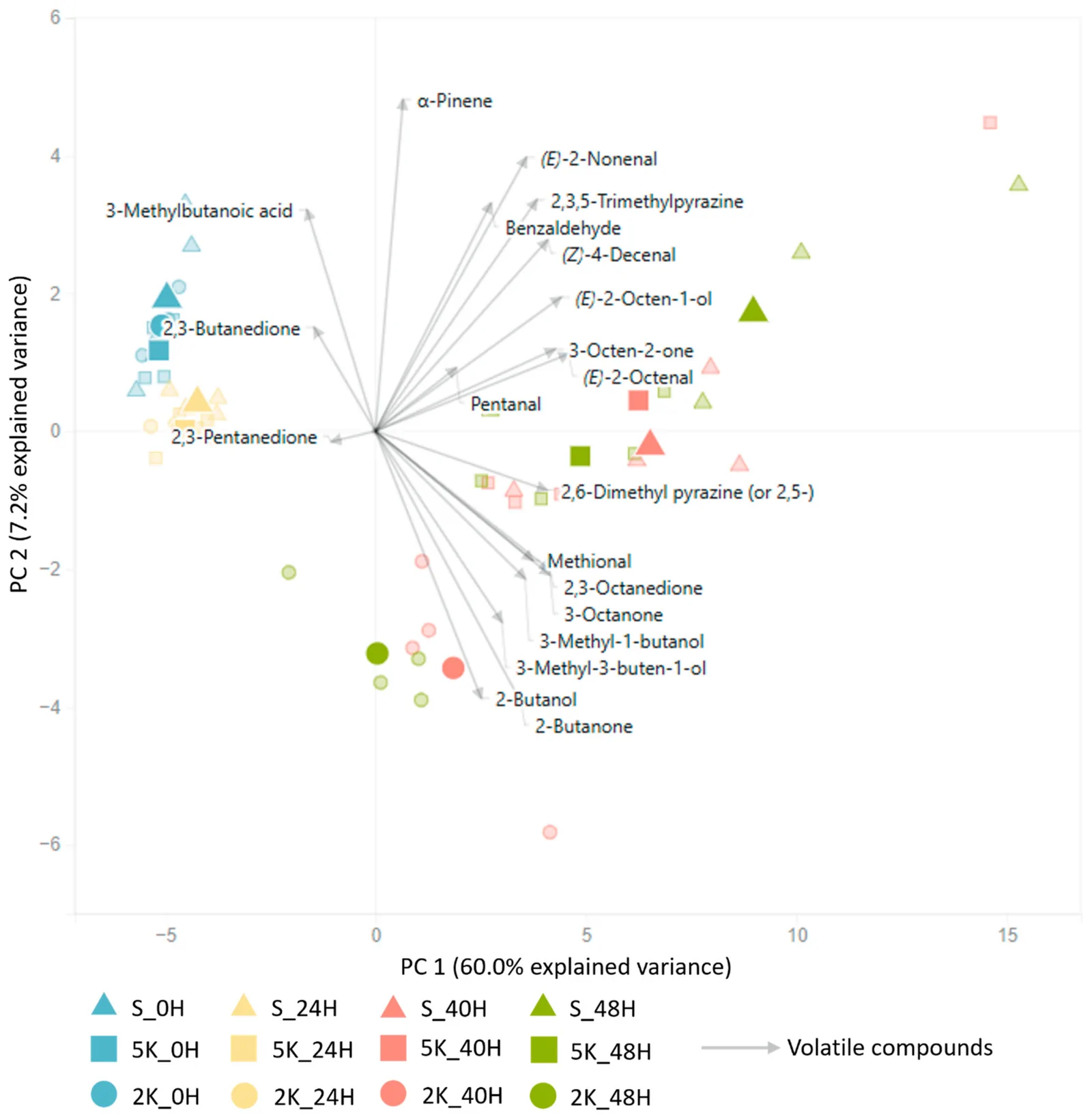
Principal component analysis (PCA) biplot showing the separation and clustering of fermented faba bean samples based on their volatile-compound profiles. The FDR-significant (*q* < 0.05) volatiles specific to the particle size × fermentation time treatment group are displayed as grey arrows. Samples fermented for 0, 24, 40, and 48 h are shown in blue, yellow, red, and green, respectively. The S (split beans), 5K (4000–5000 μm) and 2K (1000–2000 μm) particle sizes are represented by triangles, squares, and circles, respectively. Group centroids are indicated by solid symbols, whereas individual biological replicates are shown as transparent symbols in the corresponding group colour.

The PCA (Figure 2) was consistent with the trends observed in the univariate analyses, with the first two principal components explaining 67.2% of the total variance. Samples fermented for 0 and 24 h were positioned mainly on the negative side of PC1, whereas the 40 and 48 h samples were located predominantly on the positive side. The early stage samples were associated with the FDR-significant compounds 3-methylbutanoic acid, 2,3-butanedione, and 2,3-pentanedione. The later-stage samples were associated with a broader group of alcohols, aldehydes, ketones, and N-containing compounds. The upper positive-PC2 region was associated with α-pinene, (*E*)-2-nonenal, benzaldehyde, (*Z*)-4-decenal, (*E*)-2-octen-1-ol, 3-octen-2-one, (*E*)-2-octenal, and 2,3,5-trimethylpyrazine. The lower positive PC2 region was associated with 2-butanol, 2-butanone, 3-methyl-1-butanol, 3-methyl-3-buten-1-ol, methional, 2,3-octanedione, and 3-octanone. The greater dispersion of the later-stage samples indicates that particle size contributed to differences in volatile composition after 40 h of fermentation.

The combined univariate and PCA results indicate that fermentation time was the main factor shaping the volatile profile, while particle size influenced the composition of the compounds formed during the later stages. Of note, as the 2K treatment was prepared using different soaking conditions, these differences may not be attributed to particle size alone. The shift from a relatively limited early stage profile to a more diverse profile after 40 h is consistent with the development of tempeh aroma during *Rhizopus* fermentation. Feng et al. [11] also reported the formation of alcohols, ketones, aldehydes, and esters during tempeh fermentation, with the volatile profile varying according to the fermented substrate.

The early stage compounds were associated mainly with sour, sweaty, cheesy, buttery, pungent, and sweet descriptors. The decline in 3-methylbutanoic acid and 2,3-butanedione at later stages might suggest a reduced potential contribution from these early acidic and buttery notes. In contrast, the later-stage accumulation of alcohols, aldehydes, ketones, esters, furans, and pyrazines indicates the development of a more complex potential odour profile.

Specifically, the significant increase in 1-octen-3-ol, 1-octen-3-one, 3-octanol, and 3-octanone may have contributed mushroom-like, earthy, herbal, and green notes. The later increases in hexanal, heptanal, octanal, nonanal, and unsaturated aldehydes may have contributed fresh, fatty, green, waxy and cucumber-like characteristics. Esters and furans may have added fruity, sweet, winey, green, and earthy notes, while the increased pyrazines may have contributed nutty, cocoa-like, roasted, earthy, and peanut butter-like characteristics. Methional, which appeared mainly after 40 h, may have contributed musty and potato-like notes. These observations are consistent with previous descriptions of important aroma compounds in tempeh, including 1-octen-3-ol, 3-octanone, methional, and pyrazines [12].

The S and 5K treatments generally showed higher abundances of several compounds associated with mushroom-like, green, fruity, and nutty odours, corresponding to their greater visible mycelial coverage. However, the higher abundance of some alcohols in the 2K treatment, particularly 2-butanol at 48 h, indicates that particle size altered the composition of the volatile profile rather than simply increasing or decreasing overall odour intensity. Similar substrate-dependent changes in aroma have been reported for fermented faba bean ingredients produced using *R. oligosporus* [14].

### 3.5. Changes in Volatile Precursors and Associated Volatile Compounds

**Figure 3:**
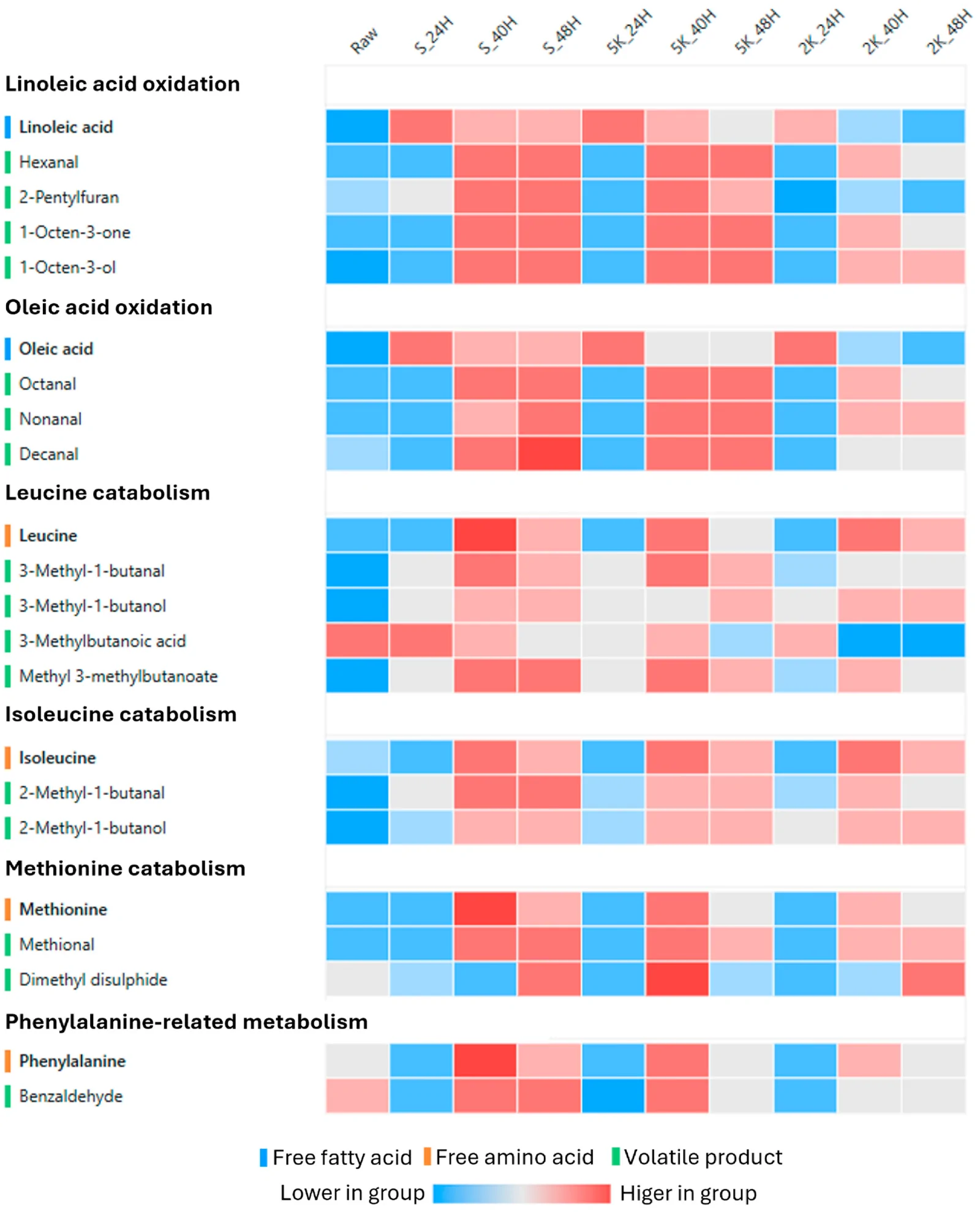
Precursor–volatile pathway profiles across fermentation treatments. Heatmap cells represent row-wise *Z*-scores of log-transformed treatment means of three biological replicates, with blue and red indicating relatively lower and higher concentrations, respectively. Precursors and their associated volatile products are grouped according to proposed metabolic pathways. Coloured row markers identify free fatty acid (blue) precursors, free amino acid precursors (orange), and volatile products (green). The Raw treatment represents faba bean powder analysed without soaking or inoculation.

The heatmap in Figure 3 illustrates the relative changes in selected volatile precursors and their associated volatile compounds during fermentation. Notably, the “raw” treatment group referred to the original faba bean samples without any treatment and was different from the time-zero treatment groups (S_0H, 5K_0H, and 2K_0H) in the previous analyses. Linoleic acid and oleic acid generally increased relative to the raw sample at 24 h, particularly in the S and 5K treatments, and subsequently declined at 40 and 48 h. This decrease was most pronounced in the 2K treatment at the later fermentation stages. In contrast, the corresponding lipid-derived volatiles accumulated mainly after 40 h. Hexanal, 2-pentylfuran, 1-octen-3-one, and 1-octen-3-ol increased in the S and 5K treatments at 40 and 48 h, whereas their relative abundance was generally lower in the 2K treatment. Similarly, octanal, nonanal, and decanal increased after 40 h, with the strongest accumulation generally observed in the S and 5K treatments.

The amino acid precursors also showed fermentation time-dependent changes. Leucine, isoleucine, methionine, and phenylalanine were relatively low in the raw and 24 h samples but increased markedly at 40 h before declining or remaining at intermediate levels at 48 h. These changes were accompanied by increases in several corresponding volatile compounds. Leucine was associated with the formation of 3-methylbutanal, 3-methyl-1-butanol, and methyl 3-methylbutanoate, which were generally more abundant at 40 and 48 h in the S and 5K treatments. In contrast, 3-methylbutanoic acid was relatively abundant in the raw and 24 h samples and declined during later fermentation. The increase in isoleucine at 40 h coincided with higher relative levels of 2-methylbutanal and 2-methyl-1-butanol. Methionine and methional showed similar patterns, with the highest relative levels generally observed at 40 h, whereas dimethyl disulphide showed a less consistent treatment-dependent pattern. Phenylalanine and benzaldehyde also increased at 40 h, particularly in the S and 5K treatments, followed by lower relative levels at 48 h.

The coordinated changes between precursor and volatile profiles are consistent with the involvement of lipid transformation and amino acid catabolism during fermentation. Lipases can release fatty acids from complex lipids, while lipoxygenases catalyse the formation of fatty acid hydroperoxides. These intermediates can subsequently undergo hydroperoxide lyase cleavage and other secondary reactions to produce aldehydes, ketones, alcohols, and furans. Faba bean has been reported to possess active lipase and lipoxygenase systems, and faba bean extracts can generate hexanal and 2-pentylfuran from linoleic acid through lipid-oxidation reactions [21,22]. However, in the present study, the faba beans were autoclaved before fermentation, which substantially reduced endogenous enzymatic activity. Therefore, the increase in linoleic- and oleic-acid-derived volatiles after 40 h, together with changes in their corresponding fatty acid precursors, is more likely attributable to microbial enzyme activity.

The amino acid patterns are also consistent with the Ehrlich pathway. In this pathway, amino acids are first converted to the corresponding *α*-keto acids by transamination, followed by decarboxylation to aldehydes and subsequent reduction or oxidation to alcohols and acids. These products may undergo further esterification to form volatile esters [23,24]. The temporal association between leucine and 3-methylbutanal, 3-methyl-1-butanol, 3-methylbutanoic acid, and methyl 3-methylbutanoate, as well as between isoleucine and its corresponding aldehyde and alcohol, supports the involvement of these transformations. The concurrent changes in methionine and methional, and in phenylalanine and benzaldehyde, are similarly consistent with S-containing and aromatic amino acid metabolism, respectively. However, because these observations were solely based on detected abundances, these patterns support, but do not validate, direct precursor-to-product conversion. The observed relationships may also be influenced by precursor release, further microbial metabolism, volatilisation, matrix interactions, and differences in extraction efficiency. In addition, our previous study found that total protein content remained largely unchanged after 48 h of fermentation, whereas sodium dodecyl sulphate–polyacrylamide gel electrophoresis and Fourier-transform infrared spectroscopy analyses indicated proteolysis and alterations in protein secondary structure [16]. These structural changes provide a possible context for the transient increases in free amino acids observed in the present study, as protein modification may increase the accessibility or release of amino acid substrates during fermentation.

### 3.6. Potential Beany Odour Reduction by Masking Effect

Fermentation has frequently been proposed as a strategy for reducing the beany characteristics of legume-based ingredients. In our previous meta-analysis of published GC–olfactometry data, the odour impacts of beany compounds, such as hexanal and 1-octen-3-ol, were significantly reduced following fermentation of pea- and soybean-based substrates [25]. Consistent with this general expectation, an informal, non-quantitative smell assessment conducted by the research team suggested that the beany odour of faba bean tempeh samples fermented for 40 h and 48 h was less pronounced than that of the raw faba bean.

Previous studies have suggested that this effect may occur through the direct transformation of beany aldehydes into less strongly perceived products. In faba bean, hexanal has been proposed to undergo reversible conversion to 1-hexanol and further oxidation to hexanoic acid, depending on the reaction conditions [26]. In a faba bean protein concentrate fermented with *Weissella confusa* A16, a decrease in hexanal accompanied by an increase in hexanoic acid ethyl ester was interpreted as being consistent with downstream hexanal degradation [27]. These studies provide recent support for the proposed aldehyde transformation mechanism, although the pathway may depend strongly on the microorganism, substrate, and fermentation conditions.

However, the results of the present study do not provide evidence for a direct removal mechanism under the fermentation conditions used. Several volatile compounds commonly associated with beany and green notes, including hexanal, octanal, nonanal, decanal, and 2-pentylfuran, increased during fermentation, particularly in the S and 5K treatments at 40 and 48 h (Table 3). Their relative abundance therefore did not show the progressive decline that would be expected if fermentation directly removed these compounds. The precursor data support this interpretation. Linoleic acid and oleic acid increased relative to the raw sample at 24 h and remained detectable during the later stages, while their associated volatile oxidation products accumulated mainly at 40 and 48 h. Although the fatty acid levels declined after 24 h, this decrease occurred alongside the formation of additional volatile products and should not be interpreted as evidence of overall precursor elimination.

The data instead suggest that fermentation promoted the production of a broad range of volatiles, including compounds associated with beany odours. The increase in lipid-derived volatiles may reflect continued oxidation, microbial transformation, or the release of compounds from the changing faba bean matrix. Likewise, the concurrent formation of amino acid-derived alcohols, aldehydes, esters, S-containing compounds, and pyrazines indicates that fermentation altered the overall volatile composition rather than simply removing the compounds associated with beaniness. A masking effect caused by newly formed compounds is therefore plausible but requires confirmation through quantitative sensory evaluation.

## 4. Conclusions

This study demonstrated that fermentation time and faba bean particle size influenced the physical and volatile characteristics of faba bean tempeh-like products fermented with *Rhizopus oryzae*. Mycelial development increased progressively, with cohesive tempeh-like structure formation evident by 40 h. The S and 5K treatments supported more extensive and uniform colonisation than the finer 2K treatment. Fermentation consistently reduced yellowness, whereas changes in lightness and redness were dependent on particle size and fermentation time. Texture showed no clear systematic differences between 40 and 48 h, indicating that much of the structural development had occurred by 40 h.

Fermentation markedly diversified the volatile profile, particularly after 40 h, with increased formation of alcohols, aldehydes, ketones, esters, furans, and N- and S-containing compounds. Particle size further influenced volatile composition during the later stages of fermentation. Changes in fatty acids and free amino acids, together with their associated volatile products, were consistent with lipid transformation and amino acid catabolism. Importantly, fermentation did not result in the direct removal of beany-associated volatiles, as several lipid-derived compounds linked to green and beany characteristics increased during fermentation. Rather, *R. oryzae* fermentation reshaped and broadened the volatile profile, potentially modifying perceived aroma through the generation of additional aroma-active compounds. Sensory evaluation is therefore required to determine whether these compositional changes translate into reduced perceived beaniness. In addition, although the soaking conditions were adjusted to achieve similar initial moisture contents, differences in soaking duration and water-to-bean ratio may independently affect the leaching of soluble compounds, enzyme activity, precursor composition and fungal growth. Therefore, the differences observed in the 2K samples may reflect both particle size and the associated soaking conditions, rather than particle size alone. Despite this limitation, the findings suggest that fermentation time and particle size are important processing variables influencing the structural development and aroma profile of faba bean products. Future microbiological characterisation at different fermentation stages could provide more insight into the contributions of *R. oryzae* and background microbiota to the observed changes.

## Figures and Tables

**Table 1:** Treatment details, soaking conditions, and final moisture contents of faba bean samples in the different treatment groups.

Treatment Group	Particle Size	Fermentation Time	Water-to-Bean Ratio (*w*/*w*) ^	Soaking Time ^	Moisture Content After Soaking (%) *
S_0H	Split	soaked and inoculated only, no incubation	1:1	6 h	50.5 ± 4.8
S_24H	Split	24 h	1:1	6 h	50.0 ± 0.7
S_40H	Split	40 h	1:1	6 h	52.4 ± 0.6
S_48H	Split	48 h	1:1	6 h	50.1 ± 1.6
5K_0H	4000–5000 μm	soaked and inoculated only, no incubation	1:1	6 h	49.8 ± 1.2
5K_24H	4000–5000 μm	24 h	1:1	6 h	48.4 ± 0.8
5K_40H	4000–5000 μm	40 h	1:1	6 h	49.3 ± 1.0
5K_48H	4000–5000 μm	48 h	1:1	6 h	46.4 ± 0.9
2K_0H	1000–2000 μm	soaked and inoculated only, no incubation	4:5	3 h	48.6 ± 0.9
2K_24H	1000–2000 μm	24 h	4:5	3 h	50.7 ± 1.3
2K_40H	1000–2000 μm	40 h	4:5	3 h	50.5 ± 0.7
2K_48H	1000–2000 μm	48 h	4:5	3 h	51.6 ± 1.5

^ Different water-to-bean ratios and soaking times were used to obtain comparable starting moisture contents across particle size samples. * Values of group mean ± standard deviation of four biological replicates.

**Table 2:** Visual and textural changes in faba bean tempeh during fermentation.

	Colour			Texture						
	L* ^t,s,ts^	a* ^t,s,ts^	b* ^t,s,ts^	Hardness (g)	Adhesiveness (g·s) ^s^	Resilience (%) ^s^	Cohesiveness ^s^	Springiness (%)	Gumminess	Chewiness
S_0H	58.1 ± 2.0 d	5.9 ± 0.8 b	32.0 ± 2.3 a	-	-	-	-	-	-	-
S_24H	56.1 ± 2.0 e	6.3 ± 0.6 a	26.6 ± 1.7 c	-	-	-	-	-	-	-
S_40H	58.0 ± 1.9 d	4.7 ± 0.7 d	20.1 ± 2.6 d	3555.2 ± 1506.7 a	−11.1 ± 7.5 a	20.5 ± 5.4 b	0.5 ± 0.1 b	52.2 ± 9.4 b	1683.4 ± 895.9 a	890.1 ± 527.3 a
S_48H	57.3 ± 3.2 de	4.1 ± 0.7 e	19.2 ± 1.3 e	4306.7 ± 1020.5 a	−15.0 ± 8.2 ab	20.3 ± 2.6 b	0.5 ± 0.1 b	56.5 ± 8.3 a	1987.4 ± 421.1 a	1120.0 ± 262.0 a
5K_0H	60.6 ± 0.9 c	5.1 ± 0.5 c	31.4 ± 1.2 a	-	-	-	-	-	-	-
5K_24H	59.4 ± 1.0 c	6.0 ± 0.4 a	26.1 ± 1.0 c	-	-	-	-	-	-	-
5K_40H	61.3 ± 1.9 b	3.9 ± 0.4 e	19.0 ± 2.2 e	3691.9 ± 1021.9 a	−9.4 ± 5.0 a	19.6 ± 1.8 b	0.5 ± 0.0 b	52.8 ± 9.0 ab	1660.3 ± 454.3 a	900.7 ± 395.5 a
5K_48H	61.4 ± 1.3 b	3.5 ± 0.2 f	18.7 ± 1.4 e	4378.3 ± 1131.6 a	−18.1 ± 5.7 b	24.4 ± 1.6 a	0.5 ± 0.0 a	52.7 ± 4.9 ab	2300.9 ± 606.1 a	1223.7 ± 371.5 a
2K_0H	64.7 ± 0.6 a	4.6 ± 0.2 d	31.8 ± 0.6 a	-	-	-	-	-	-	-
2K_24H	62.6 ± 0.9 b	5.5 ± 0.2 b	29.7 ± 1.0 b	-	-	-	-	-	-	-
2K_40H	61.5 ± 1.7 b	4.2 ± 0.3 e	19.5 ± 1.2 de	3318.8 ± 1088.8 a	−19.1 ± 11.2 b	22.9 ± 1.8 a	0.5 ± 0.0 a	58.2 ± 5.0 a	1729.4 ± 616.5 a	1030.2 ± 482.7 a
2K_48H	56.5 ± 2.1 e	4.8 ± 0.5 cd	20.8 ± 1.9 d	3683.8 ± 798.1 a	−11.9 ± 4.3 a	20.6 ± 1.7 b	0.5 ± 0.0 b	49.6 ± 3.3 b	1769.9 ± 395.7 a	880.7 ± 220.0 a

Note: Colour measurements are expressed using the CIE L*, a*, and b* scales. Since a firm tempeh structure suitable for texture analysis was observed only in the 40H and 48H treatment groups, the remaining groups were not analysed using the texture analyser. Group means of four biological replicates for individual colour and textural parameters were compared using one-way ANOVA followed by Fisher’s LSD post hoc test. Values are expressed as mean ± standard deviation. Significant differences (*p* < 0.05) are indicated by different lowercase letters. Parameters were significantly affected by fermentation time, particle size, and their interaction (*q* < 0.05; Benjamini–Hochberg FDR correction), denoted by the superscripts t, s, and ts, respectively.

**Table 3:** Peak responses (×10^−5^) of volatile compounds in faba bean samples fermented for different durations using different particle sizes.

		Split					5K Particle Size					2K Particle Size			
	Odour Descriptor	S_0H	S_24H	S_40H	S_48H		5K_0H	5K_24H	5K_40H	5K_48H		2K_0H	2K_24H	2K_40H	2K_48H
								**Acids**							
3-Methylbutanoic acid ^t,ts^	sour, sweaty, cheesy	48.5 ± 56.1 abcd	100.6 ± 57.3 ab	58.3 ± 35.6 abc	20.1 ± 23.6 cde		46.4 ± 52.1 abcd	26.6 ± 22.5 bcd	32.8 ± 37.8 cde	10.6 ± 13.0 de		157.6 ± 79.1 a	42.5 ± 18.6 abcd	0.0 ± 0.0 e	0.0 ± 0.0 e
								**Alcohols**							
2-Butanol ^t^	sweet, apricot	130.5 ± 54.8 d	164.1 ± 54.2 d	736.4 ± 226.8 b	855.6 ± 274.8 b		152.0 ± 38.0 d	472.8 ± 639.5 cd	1226.6 ± 1173.6 b	740.5 ± 430.0 bc		144.1 ± 68.1 d	165.3 ± 53.4 d	1482.3 ± 672.0 ab	2298.2 ± 386.0 a
3-Methyl-3-buten-1-ol ^t^	herbaceous	0.0 ± 0.0 e	178.7 ± 176.6 cd	370.8 ± 176.4 abc	595.9 ± 67.4 ab		5.1 ± 6.3 e	172.9 ± 208.3 d	339.5 ± 71.0 abc	956.5 ± 288.6 a		0.0 ± 0.0 e	280.7 ± 224.9 bcd	500.1 ± 133.6 ab	352.5 ± 206.0 abc
3-Methyl-1-butanol ^t,s^	fusel, alcoholic, whiskey	0.0 ± 0.0 g	1165.7 ± 283.8 ef	2271.4 ± 459.3 bc	4240.0 ± 1119.6 a		0.0 ± 0.0 g	920.3 ± 295.5 f	1362.0 ± 493.8 def	1585.4 ± 183.2 cde		0.0 ± 0.0 g	942.6 ± 309.4 f	2000.4 ± 426.4 bcd	2861.0 ± 1617.8 b
2-Methyl-1-butanol ^t^	ethereal, fusel, alcoholic	0.0 ± 0.0 d	222.6 ± 229.4 c	1691.5 ± 325.6 a	1660.3 ± 439.5 a		0.0 ± 0.0 d	182.6 ± 97.5 bc	910.5 ± 330.1 a	1058.6 ± 338.5 a		0.0 ± 0.0 d	326.3 ± 74.7 b	1208.8 ± 378.8 a	2172.0 ± 1693.5 a
1-Pentanol ^t^	fusel, oily, sweet	0.0 ± 0.0 d	54.9 ± 25.9 b	91.4 ± 16.7 a	116.6 ± 33.8 a		0.0 ± 0.0 d	37.1 ± 14.2 bc	99.7 ± 15.9 a	133.4 ± 31.6 a		0.0 ± 0.0 d	32.4 ± 17.7 c	133.9 ± 26.3 a	111.2 ± 19.3 a
1-Hexanol ^t,ts^	ethereal, fusel, oily	69.2 ± 26.8 ef	175.1 ± 76.0 cd	336.2 ± 71.0 bc	645.3 ± 321.9 ab		53.0 ± 32.5 f	121.5 ± 45.6 de	316.3 ± 92.6 bc	554.6 ± 135.4 ab		109.9 ± 138.0 ef	48.4 ± 20.6 f	642.3 ± 202.6 ab	1004.7 ± 260.5 a
1-Octen-3-ol ^t^	mushroom, earthy, green	15.0 ± 5.5 e	148.9 ± 94.9 c	24,897.0 ± 4279.7 a	31,145.0 ± 9016.8 a		15.0 ± 14.1 e	119.5 ± 95.9 cd	24,778.0 ± 14,594.0 ab	23,914.0 ± 6185.0 a		26.7 ± 10.0 de	95.6 ± 53.9 c	13,056.0 ± 1187.8 ab	8107.4 ± 2183.4 b
3-Octanol ^t^	earthy, mushroom, herbal	3.9 ± 4.2 bc	6.3 ± 3.6 b	74.0 ± 17.6 a	74.8 ± 29.4 a		3.1 ± 1.0 bc	3.3 ± 2.3 bc	90.8 ± 54.5 a	56.3 ± 3.5 a		2.7 ± 1.9 c	4.2 ± 2.2 bc	77.9 ± 15.2 a	44.1 ± 9.0 a
2-Ethyl-1-hexanol ^t^	citrus, fresh, floral	68.5 ± 37.2 b	97.0 ± 31.1 b	292.1 ± 53.6 a	383.2 ± 136.8 a		96.1 ± 38.4 b	63.0 ± 23.3 b	360.2 ± 98.9 a	377.6 ± 48.0 a		91.2 ± 65.4 b	65.9 ± 23.2 b	315.2 ± 83.9 a	289.1 ± 41.8 a
(*E*)-2-Octen-1-ol ^t,s,ts^	green, citrus, vegetable	0.0 ± 0.0 d	0.0 ± 0.0 d	1436.4 ± 730.4 a	1928.1 ± 779.5 a		0.0 ± 0.0 d	0.0 ± 0.0 d	1583.2 ± 1442.0 a	1492.3 ± 727.4 a		0.0 ± 0.0 d	0.0 ± 0.0 d	514.0 ± 85.1 b	193.5 ± 71.0 c
1-Octanol ^t,s^	waxy, green, orange	7.5 ± 6.4 de	7.4 ± 2.1 d	675.6 ± 271.9 ab	992.2 ± 533.3 a		6.5 ± 3.5 de	5.5 ± 3.7 de	777.2 ± 639.0 ab	624.5 ± 260.0 ab		4.6 ± 3.8 de	2.7 ± 2.1 e	327.8 ± 49.8 bc	216.3 ± 72.0 c
								**Aldehydes**							
3-methylbutanal ^t,s,ts^	ethereal, aldehydic, chocolate	138.1 ± 77.6 g	4010.5 ± 891.6 f	35,038.0 ± 10,442.0 a	15,055.0 ± 6170.9 bc		137.1 ± 57.2 g	3578.4 ± 1218.4 f	15,984.0 ± 1828.6 b	8254.3 ± 1046.0 cd		106.8 ± 31.6 g	3023.4 ± 1458.4 f	7398.6 ± 2159.0 de	4333.5 ± 1077.8 ef
2-methylbutanal ^t,s^	musty, cocoa, phenolic	119.0 ± 59.9 f	2883.5 ± 696.3 d	25,092.0 ± 3578.9 a	10,909.0 ± 2400.9 b		96.5 ± 38.1 f	1445.9 ± 1091.3 e	8976.1 ± 4360.3 bc	5471.3 ± 1574.9 bcd		70.1 ± 32.4 f	1545.7 ± 1440.2 e	5000.8 ± 2084.6 cd	3118.8 ± 816.0 d
Pentanal ^s^	fermented, bready, fruity	106.4 ± 51.5 abc	23.7 ± 17.1 bcd	0.0 ± 0.0 d	13.6 ± 18.3 cd		116.7 ± 30.7 ab	680.3 ± 729.1 ab	5424.6 ± 7381.6 a	1400.6 ± 2064.8 ab		99.9 ± 29.7 abc	1124.0 ± 1656.2 ab	2224.8 ± 1495.0 a	851.8 ± 997.7 ab
Hexanal ^t,s,ts^	fresh, green, fatty	1007.8 ± 565.9 e	1798.3 ± 491.6 c	10,867.0 ± 2044.4 a	11,785.0 ± 1786.8 a		1141.5 ± 374.9 de	1538.0 ± 355.1 cd	10,316.0 ± 1367.0 a	11,486.0 ± 2700.6 a		1083.4 ± 262.8 de	1581.2 ± 634.3 cd	6534.1 ± 963.2 b	4409.0 ± 1074.7 b
Heptanal ^t,s^	fresh, aldehydic, fatty	31.1 ± 15.3 e	95.0 ± 35.5 d	452.2 ± 58.0 ab	573.4 ± 204.9 a		35.6 ± 13.3 e	81.7 ± 29.2 d	476.7 ± 172.4 a	472.6 ± 84.0 a		35.1 ± 15.5 e	83.9 ± 44.6 d	267.2 ± 80.1 bc	217.2 ± 66.1 c
(*E*,*E*)-2,4-Hexadienal ^t^	sweet, green, spicy	10.7 ± 6.7 e	31.3 ± 13.7 d	147.6 ± 19.1 ab	191.5 ± 64.9 a		10.1 ± 4.6 e	29.2 ± 10.5 d	148.6 ± 58.1 ab	157.9 ± 33.0 ab		12.2 ± 6.0 e	28.6 ± 17.0 d	85.5 ± 23.7 bc	72.0 ± 27.1 c
(*Z*)-2-Heptenal (or (*E*)-) ^t^	green, fatty	43.0 ± 44.1 cde	7.6 ± 7.8 e	106.4 ± 13.7 a	118.4 ± 38.2 a		13.0 ± 9.0 de	24.5 ± 26.1 cde	101.6 ± 43.6 a	76.3 ± 11.7 ab		24.6 ± 5.7 bcd	15.2 ± 11.0 cde	41.3 ± 15.0 abc	35.5 ± 19.4 abcd
Benzaldehyde ^t^	sharp, sweet, bitter	204.2 ± 67.8 ab	65.3 ± 25.6 c	290.1 ± 74.0 a	217.8 ± 110.4 ab		188.5 ± 101.5 ab	43.4 ± 24.4 c	237.9 ± 178.6 ab	134.8 ± 26.7 b		208.0 ± 56.5 ab	45.2 ± 21.1 c	148.1 ± 52.7 b	128.4 ± 40.7 b
Octanal ^t,s^	aldehydic, waxy, citrus	40.5 ± 29.6 de	40.3 ± 21.2 d	916.3 ± 259.8 a	1266.8 ± 688.7 a		14.3 ± 6.5 e	28.3 ± 20.1 de	924.8 ± 778.4 a	654.7 ± 169.0 ab		17.4 ± 7.1 de	26.1 ± 16.6 de	308.4 ± 76.6 bc	157.5 ± 49.4 c
(*E*)-2-Octenal ^t,s,ts^	fresh, cucumber, fatty	0.0 ± 0.0 d	0.0 ± 0.0 d	1790.4 ± 668.2 a	2230.5 ± 575.1 a		0.0 ± 0.0 d	0.0 ± 0.0 d	1815.6 ± 1205.2 a	1834.1 ± 539.6 a		0.0 ± 0.0 d	0.0 ± 0.0 d	769.9 ± 145.4 b	419.5 ± 130.1 c
Nonanal ^t,ts^	waxy, aldehydic, rose	40.8 ± 15.1 ef	76.6 ± 37.6 e	968.2 ± 305.0 bc	2252.3 ± 1241.7 a		39.8 ± 15.1 ef	42.4 ± 34.5 f	1321.7 ± 1106.2 ab	1342.2 ± 281.3 ab		35.6 ± 13.1 f	69.7 ± 23.8 ef	537.0 ± 114.9 cd	475.4 ± 132.5 d
(*E*)-2-Nonenal ^t,s,ts^	fatty, green, cucumber	9.1 ± 7.1 de	5.3 ± 2.1 de	15.2 ± 16.0 cd	104.8 ± 70.4 a		5.1 ± 1.4 de	4.6 ± 2.6 de	54.4 ± 82.3 bc	38.7 ± 22.5 ab		5.7 ± 2.1 de	3.8 ± 1.1 de	4.1 ± 0.5 de	3.2 ± 0.8 e
(Z)-4-Decenal ^t,s,ts^	citrus, aldehydic, orange	31.0 ± 22.5 e	20.6 ± 7.7 e	483.4 ± 315.4 bc	2073.0 ± 1133.9 a		23.3 ± 7.4 e	14.7 ± 7.6 e	1209.0 ± 1176.6 ab	1102.2 ± 511.9 a		25.8 ± 10.6 e	17.2 ± 5.7 e	149.6 ± 93.4 d	274.4 ± 106.9 cd
Decanal ^t^	sweet, aldehydic, waxy	59.4 ± 51.9 cde	32.5 ± 14.9 de	142.8 ± 80.7 ab	287.9 ± 168.8 a		36.9 ± 16.3 cde	23.4 ± 15.5 e	163.6 ± 87.3 ab	147.3 ± 33.4 ab		40.0 ± 20.8 cde	23.9 ± 9.4 e	85.1 ± 47.6 bc	67.0 ± 28.9 bcd
								**Ketones**							
2,3-Butanedione ^t^	buttery, sweet, creamy	152.4 ± 222.6 abc	116.5 ± 46.8 ab	47.5 ± 37.8 cde	27.1 ± 11.5 de		61.6 ± 7.2 bcd	110.0 ± 37.4 ab	66.6 ± 43.2 bcd	21.1 ± 8.6 e		70.6 ± 44.7 bcd	184.6 ± 120.3 a	45.9 ± 15.1 bcde	88.0 ± 23.1 abc
2-Butanone ^t^	acetone, ethereal, fruity	181.0 ± 126.2 d	1753.6 ± 440.2 c	3487.8 ± 879.5 a	3841.7 ± 1357.7 a		152.1 ± 23.2 d	1963.3 ± 688.8 bc	3682.7 ± 680.1 a	3406.2 ± 1515.6 ab		190.6 ± 70.9 d	1855.2 ± 530.0 c	4298.0 ± 747.9 a	3881.5 ± 809.8 a
3-Pentanone ^t,s,ts^	ethereal, acetone	123.8 ± 62.8 g	3071.6 ± 851.6 e	27,186.0 ± 4057.0 a	12,339.0 ± 3048.5 b		119.9 ± 45.7 g	2495.3 ± 1039.7 ef	12,466.0 ± 1244.9 b	6766.7 ± 509.9 c		106.5 ± 33.1 g	2189.9 ± 2042.6 f	5913.4 ± 1680.8 cd	3426.7 ± 815.4 de
2,3-Pentanedione ^t^	pungent, sweet, buttery	18.8 ± 8.5 cde	26.8 ± 5.2 ab	20.8 ± 2.2 bcd	15.6 ± 5.2 de		19.1 ± 6.3 bcde	24.3 ± 6.9 abc	18.3 ± 1.9 bcde	13.3 ± 2.0 e		17.6 ± 5.8 cde	32.7 ± 8.1 a	23.4 ± 5.6 abc	17.5 ± 3.8 cde
2,3-Hexanedione ^t,ts^	dry, sweet, caramellic	0.0 ± 0.0 f	59.7 ± 16.7 b	18.6 ± 3.8 cd	32.9 ± 20.9 c		0.0 ± 0.0 f	54.9 ± 21.5 b	23.3 ± 7.7 cd	21.8 ± 14.3 cd		0.0 ± 0.0 f	109.8 ± 23.2 a	14.3 ± 6.2 de	9.5 ± 4.8 e
2-Heptanone ^t,ts^	fruity, spicy, sweet	50.4 ± 33.3 e	84.4 ± 38.9 de	184.2 ± 42.2 abc	284.9 ± 126.1 a		53.2 ± 13.0 e	50.2 ± 18.0 e	147.5 ± 32.0 abcd	221.9 ± 81.9 ab		53.7 ± 26.4 e	138.0 ± 41.8 bcd	127.8 ± 43.7 bcd	103.7 ± 35.9 cd
1-Octen-3-one ^t,s,ts^	herbal, mushroom, earthy	0.0 ± 0.0 e	0.0 ± 0.0 e	1729.0 ± 739.2 b	2638.1 ± 891.2 a		0.0 ± 0.0 e	0.0 ± 0.0 e	2908.7 ± 2811.0 ab	2711.9 ± 439.1 a		0.0 ± 0.0 e	0.0 ± 0.0 e	519.1 ± 122.7 c	172.7 ± 61.5 d
3-Octanone ^t^	fresh, herbal, lavender	8.4 ± 3.1 e	54.1 ± 24.4 d	9741.9 ± 1432.2 a	5513.7 ± 1318.3 b		7.3 ± 2.8 e	41.7 ± 12.6 d	9507.9 ± 3080.8 a	6415.0 ± 721.2 ab		11.6 ± 4.9 e	58.3 ± 36.0 d	8965.3 ± 938.0 ab	3065.6 ± 788.1 c
6-Methyl-5-hepten-2-one ^t^	citrus, green, musty	0.0 ± 0.0 e	33.3 ± 17.0 c	794.1 ± 175.3 a	663.0 ± 51.2 a		0.0 ± 0.0 e	22.4 ± 17.6 d	788.4 ± 235.8 a	759.4 ± 153.5 a		0.0 ± 0.0 e	24.4 ± 15.3 cd	597.9 ± 115.1 ab	264.2 ± 77.0 b
2,3-Octanedione ^t,ts^	dill, asparagus, cilantro	0.0 ± 0.0 f	20.5 ± 8.7 e	4062.1 ± 736.4 a	2293.6 ± 553.3 c		0.0 ± 0.0 f	0.0 ± 0.0 f	4113.8 ± 1335.9 a	2658.3 ± 298.6 bc		0.0 ± 0.0 f	22.7 ± 14.6 e	3738.7 ± 366.5 ab	1266.9 ± 258.5 d
3-Octen-2-one ^t,ts^	earthy, spicy, herbal	2.6 ± 3.0 d	1.2 ± 1.5 d	22.5 ± 8.3 b	48.7 ± 9.2 a		0.6 ± 0.7 d	0.5 ± 0.8 d	34.1 ± 20.3 ab	49.3 ± 15.3 a		0.8 ± 0.5 d	2.1 ± 3.1 d	14.3 ± 2.4 bc	9.3 ± 3.5 c
2-Decanone ^t^	orange, floral, fatty	7.7 ± 6.8 bc	3.8 ± 2.5 c	32.4 ± 26.1 ab	88.6 ± 55.2 a		4.6 ± 1.8 c	3.5 ± 1.5 c	31.3 ± 31.7 a	37.3 ± 19.2 a		5.4 ± 2.5 c	3.7 ± 1.7 c	39.2 ± 28.4 a	48.1 ± 17.2 a
								**Esters**							
Methyl 3-methylbutanoate ^t,s^	apple, fruity, pineapple	5.8 ± 3.1 f	495.8 ± 104.9 d	2956.2 ± 702.8 a	2373.2 ± 1081.5 ab		6.6 ± 3.3 f	416.5 ± 180.5 de	3057.2 ± 1424.4 a	1003.0 ± 274.6 c		4.4 ± 2.2 f	263.6 ± 152.7 e	1209.4 ± 281.2 bc	722.3 ± 160.2 cd
Methyl heptanoate ^t,s,ts^	sweet, fruity, green	0.0 ± 0.0 e	1.8 ± 1.0 d	71.8 ± 9.4 ab	95.1 ± 37.9 a		0.0 ± 0.0 e	0.5 ± 0.6 e	72.1 ± 27.1 ab	53.6 ± 14.1 bc		0.0 ± 0.0 e	1.6 ± 1.0 d	38.1 ± 9.9 c	35.8 ± 12.2 c
								**Furans**							
2-Butylfuran ^t,s,ts^	fruity, winey, sweet	2.9 ± 1.2 f	5.1 ± 1.0 e	39.0 ± 10.8 ab	47.3 ± 20.6 a		2.8 ± 0.7 f	3.8 ± 1.0 ef	29.7 ± 12.6 bc	36.7 ± 8.6 ab		2.3 ± 1.0 f	6.3 ± 2.2 e	22.8 ± 5.8 c	13.3 ± 4.7 d
2-Pentylfuran ^t,s,ts^	fruity, green, earthy	34.2 ± 14.8 cd	39.4 ± 12.5 bc	84.5 ± 14.8 a	119.5 ± 57.4 a		27.9 ± 9.0 cde	22.0 ± 8.7 def	82.0 ± 37.3 a	67.3 ± 3.6 ab		25.4 ± 11.1 cde	13.0 ± 4.7 f	29.2 ± 6.2 cde	19.3 ± 9.5 ef
								**S-containing compounds**							
Dimethyl disulphide	sulphurous, vegetable, cabbage	4.7 ± 2.7 a	6.3 ± 3.3 a	3.9 ± 0.7 a	35.8 ± 60.9 a		7.7 ± 4.5 a	4.1 ± 1.1 a	204.1 ± 397.5 a	6.2 ± 1.5 a		28.3 ± 43.8 a	3.8 ± 2.0 a	4.5 ± 1.7 a	52.0 ± 92.1 a
Methional ^t^	musty, potato, tomato	0.0 ± 0.0 d	0.0 ± 0.0 d	20.7 ± 13.4 a	14.0 ± 7.1 ab		0.0 ± 0.0 d	0.0 ± 0.0 d	15.1 ± 1.6 ab	6.4 ± 3.4 c		0.0 ± 0.0 d	0.0 ± 0.0 d	7.4 ± 5.5 c	11.1 ± 6.7 bc
								**N-containing compounds**							
Methylpyrazine ^t^	nutty, cocoa, roasted	10.4 ± 2.9 fg	26.0 ± 8.5 cd	86.9 ± 41.6 ab	167.5 ± 125.8 a		12.0 ± 6.7 efg	18.5 ± 5.9 def	65.1 ± 28.7 b	44.8 ± 11.5 bc		8.9 ± 2.3 g	22.1 ± 7.6 de	71.9 ± 33.1 ab	54.7 ± 14.2 b
2,6-dimethylpyrazine (or 2,5-) ^t^	ethereal, cocoa, nutty	51.8 ± 17.2 ef	72.2 ± 25.6 de	340.7 ± 112.4 a	233.0 ± 56.6 ab		43.7 ± 19.4 f	57.9 ± 16.1 ef	383.5 ± 140.2 a	161.4 ± 35.0 bc		41.4 ± 14.7 f	67.9 ± 18.9 e	271.4 ± 45.6 a	110.5 ± 29.8 cd
2,3,5-Trimethylpyrazine ^t,s^	nutty, earthy, powdery, cocoa	16.3 ± 8.5 de	21.1 ± 5.3 cde	224.3 ± 77.4 a	261.5 ± 234.4 ab		9.3 ± 2.7 de	13.6 ± 4.1 de	173.2 ± 237.8 abc	90.1 ± 99.7 bcd		10.3 ± 2.4 de	12.0 ± 3.4 de	14.1 ± 23.0 e	31.8 ± 5.9 cd
								**Terpenes**							
α-Pinene ^s^	fresh, camphoreous, pine	17.9 ± 14.7 ab	9.5 ± 4.3 ab	8.1 ± 4.0 abc	17.2 ± 10.5 a		8.3 ± 5.0 abc	7.3 ± 3.5 bc	7.3 ± 4.2 bc	7.4 ± 3.3 abc		7.9 ± 3.9 abc	2.2 ± 1.5 d	3.4 ± 0.6 cd	1.7 ± 0.5 d
β-Ocimene	citrus, tropical, green	3.9 ± 1.8 a	2.3 ± 0.8 a	3.5 ± 0.9 a	5.2 ± 0.7 a		4.0 ± 2.3 a	2.8 ± 1.9 a	3.4 ± 1.4 a	3.9 ± 1.5 a		7.6 ± 5.1 a	2.9 ± 1.7 a	45.2 ± 87.1 a	1.9 ± 0.6 a
D-Limonene	citrus, orange, fresh	6.9 ± 3.2 a	6.0 ± 1.8 a	8.6 ± 6.0 a	14.7 ± 7.5 a		7.7 ± 3.9 a	5.1 ± 3.0 a	11.2 ± 4.3 a	11.0 ± 3.4 a		10.1 ± 7.5 a	5.9 ± 3.4 a	68.5 ± 126.8 a	6.0 ± 2.2 a
								**Hydrocarbon**							
Ethylbenzene ^t^	Ethereal, sweet	7.6 ± 3.3 e	9.7 ± 1.3 bcde	16.2 ± 4.3 a	14.0 ± 4.1 ab		7.6 ± 0.9 de	8.7 ± 3.0 cde	14.4 ± 5.1 ab	12.3 ± 1.3 abc		11.0 ± 4.4 abcde	8.4 ± 3.1 cde	12.1 ± 1.7 abc	11.9 ± 4.6 abcd

Note: Means of four biological replicates for individual volatile compounds were compared using one-way ANOVA followed by Fisher’s LSD post hoc test. Values are presented as mean ± standard deviation. Different lowercase letters indicate significant differences (*p* < 0.05). Volatile compounds were significantly affected by fermentation time, particle size, and their interaction (*q* < 0.05; Benjamini–Hochberg FDR correction), denoted by the superscripts t, s, and ts, respectively. Odour descriptors were obtained from The Good Scents Company and VCF databases.

## Data Availability

The data supporting the findings of this study are included in the article and its Supplementary Materials. Further information is available from the corresponding authors upon reasonable request.

## Supplementary Materials

The following supporting information can be downloaded at: https://www.mdpi.com/article/10.3390/foods15203683/s1. The Supplementary Materials submitted with this manuscript include Supplementary Table S1, Volatile compounds identified in faba bean samples, and Supplementary Table S2, Free amino acid and free fatty acid contents of faba bean samples.
