# Supplementary material for: Physical, Volatile, and Flavour Precursor Changes in Faba Bean During Rhizopus oryzae Fermentation
Source: Foods. 2026 Oct 16;15(20):3683. doi: 10.3390/foods15203683 (PMC13704464; doi:10.3390/foods15203683)
Supplement: Supplementary file 1 [file foods-15-03683-s001.zip › foods-4599954-supplementary.pdf]

Supplementary Table S1. Volatile compounds identified in faba bean samples.

Compound	CAS	Calculated retention index	Reference retention index (semi-polar)	Target ion (*m/z*)	Qualifier 1 (*m/z*)	Qualifier 2 (*m/z*)	Qualifier 3 (*m/z*)
2,3-Butanedione	431-03-8	<700	595	86	42		
2-Butanone	78-93-3	<700	598	72	57		
2-Butanol	78-92-2	<700	598	45	59		
3-Methyl-1-butanal	590-86-3	<700	652	44	58	71	
2-Methyl-1-butanal	96-17-3	<700	662	57	41	86	
3-Pentanone	96-22-0	<700	688	57	86		
Pentanal	110-62-3	703.4424	700	44	58		
2,3-Pentanedione	600-14-6	703.4424	697	57	100		
3-Methyl-3-buten-1-ol	763-32-6	732.0626	731	41	56	86	
3-Methyl-1-butanol	123-51-3	737.4111	736	55	41	70	
2-Methyl-1-butanol	137-32-6	740.4552	739	41	57	70	
Dimethyl disulphide	624-92-0	744.5519	746	94	45		
1-Pentanol	71-41-0	771.4737	765	42	55	70	
Methyl 3-methylbutanoate	556-24-1	778.3499	776	74	59	85	101
2,3-Hexanedione	3848-24-6	791.7297	786	71	114		
Hexanal	66-25-1	805.2658	801	56	44	72	82
Methylpyrazine	109-08-0	834.2684	829	94	67	53	
3-Methylbutanoic acid	503-74-2	871.0278	850	60	87		
Ethylbenzene	100-41-4	872.4101	855	91	106	51	
1-Hexanol	111-27-3	875.9747	868	56	41	69	
2-Butylfuran	4466-24-4	892.8506	893	81	53	124	
2-Heptanone	110-43-0	895.2658	891	43	58	71	
Heptanal	111-71-7	906.0762	901	44	70	55	
(*E*,*E*)-2,4-Hexadienal	142-83-6	906.8863	911	81	39	96	
Methional	3268-49-3	915.0689	907	48	104	76	
2,6-Dimethyl pyrazine (or 2,5-)	108-50-9	919.2006	917	108	42	67	
α-Pinene	80-56-8	933.8887	937	93	77	121	
(*Z*)-2-Heptenal (or (*E*)-)	57266-86-1	962.8679	958	41	55	83	112

Benzaldehyde	100-52-7	971.1045	962	77	105	51	
1-Octen-3-one	4312-99-6	980.7075	979	55	70	83	
1-Octen-3-ol	3391-86-4	983.8779	980	57	43	72	85
3-Octanone	106-68-3	988.5768	986	43	57	72	99
6-Methyl-5-hepten-2-one	110-93-0	989.414	986	55	69	108	
2,3-Octanedione	585-25-1	990.0621	984	99	71	142	
2-Pentylfuran	3777-69-3	992.0335	993	81	138		
3-Octanol	589-98-0	999.7056	993	59	55	83	101
Octanal	124-13-0	1006.494	1003	41	57	84	69
2,3,5-Trimethylpyrazine	14667-55-1	1009.425	1004	122	81	54	
Methyl heptanoate	106-73-0	1026.273	1023	74	87	55	113
ß-Ocimene	13877-91-3	1031.314	1037	93	91	105	121
D-Limonene	5989-27-5	1031.689	1031	68	93	136	121
2-Ethyl-1-hexanol	104-76-7	1032.857	1030	57	41	70	83
3-Octen-2-one	1669-44-9	1043.756	1040	55	111	126	
(*E*)-2-Octenal	2548-87-0	1063.718	1060	41	55	70	83
(*E*)-2-Octen-1-ol	18409-17-1	1072.029	1067	57	41	67	110
1-Octanol	111-87-5	1074.412	1070	56	69	84	
Nonanal	124-19-6	1107.837	1104	57	41	70	98
(*E*)-2-Nonenal	18829-56-6	1165.431	1162	55	70	83	96
2-Decanone	693-54-9	1194.595	1193	58	71	59	
(*Z*)-4-Decenal	21662-09-9	1198.797	1193	41	55	84	110
Decanal	112-31-2	1210.8	1206	57	70	112	128

Note: reference retention indices were obtained from the NIST 20 library for semi-polar columns (VF-5ms is classified as a semi-polar column).

Supplementary Table S2. Free amino acid and free fatty acid contents of faba bean samples.

			Split			5K particle size			2K particle size	
	Raw (n=3)	S_24H (n=3)	S_40H (n=3)	S_48H (n=3)	5K_24H (n=3)	5K_40H (n=3)	5K_48H (n=3)	2K_24H (n=3)	2K_40H (n=3)	2K_48H (n=3)
**Moisture content (%)**	8.33 ± 1.12d	49.85 ± 0.74b	52.46 ± 0.67a	49.40 ± 1.01b	48.73 ± 0.68b	49.22 ± 1.16b	46.44 ± 1.05c	50.42 ± 1.38b	50.68 ± 0.76a	51.83 ± 1.78a
**Amino acids (µg/g)**										

Glycine	10.57 ± 0.68 cd	2.03 ± 0.62 d	29.72 ± 12.38 a	13.00 ± 1.65 bc	1.63 ± 0.45 d	18.54 ± 8.76 bc	12.58 ± 1.39 bc	2.35 ± 0.64 d	21.15 ± 9.90 ab	15.07 ± 4.65 bc
Serine	1.53 ± 0.04 c	2.01 ± 0.39 c	17.35 ± 7.00 a	8.28 ± 0.89 b	2.02 ± 0.24 c	11.27 ± 5.06 b	8.66 ± 1.37 b	2.20 ± 0.38 c	13.44 ± 5.50 ab	12.87 ± 4.08 ab
Methionine	0.57 ± 0.04 cd	0.48 ± 0.11 cd	6.28 ± 3.10 a	2.22 ± 0.51 bcd	0.41 ± 0.09 d	3.11 ± 1.75 b	1.62 ± 0.21 bcd	0.51 ± 0.15 cd	2.49 ± 1.12 bc	1.63 ± 0.44 bcd
Proline	14.96 ± 1.32 ab	1.44 ± 0.42 c	20.84 ± 10.29 a	9.28 ± 1.76 bc	1.50 ± 1.12 c	12.41 ± 7.27 ab	7.29 ± 1.32 bc	0.91 ± 0.20 c	14.84 ± 8.22 ab	11.45 ± 3.95 b
Leucine	6.96 ± 1.13 c	4.70 ± 0.95 c	63.66 ± 32.43 a	23.89 ± 4.73 bc	4.78 ± 1.09 c	33.16 ± 18.12 b	18.38 ± 2.40 bc	5.87 ± 1.11 c	28.96 ± 14.48 b	19.38 ± 6.65 bc
Creatine	0.00 ± 0.00 a	0.00 ± 0.00 a	0.00 ± 0.00 a	0.00 ± 0.00 a	0.00 ± 0.00 a	0.00 ± 0.00 a	0.00 ± 0.00 a	0.00 ± 0.00 a	0.00 ± 0.00 a	0.00 ± 0.00 a
Glutamic acid	159.04 ± 6.74 abc	54.82 ± 22.88 c	295.88 ± 173.34 ab	139.41 ± 53.85 bc	50.52 ± 23.70 c	200.36 ± 132.33 abc	175.16 ± 73.66 abc	40.11 ± 19.16 c	326.74 ± 194.47 ab	334.48 ± 175.30 a
Phenylalanine	20.63 ± 1.49 b	4.22 ± 0.64 de	35.12 ± 16.09 a	13.47 ± 2.25 bcde	3.68 ± 0.83 e	17.51 ± 8.75 bc	9.92 ± 0.81 bcde	4.07 ± 0.44 de	14.54 ± 6.65 bcd	9.85 ± 3.00 cde
Lysine	0.19 ± 0.02 c	1.97 ± 0.42 c	11.20 ± 2.84 b	7.65 ± 1.42 b	2.05 ± 0.38 c	9.27 ± 2.82 b	7.09 ± 1.82 b	2.09 ± 0.57 c	15.86 ± 4.47 a	17.58 ± 5.51 a
Aminobutyric acid	0.32 ± 0.00 a	0.72 ± 0.20 a	1.13 ± 0.35 a	0.70 ± 0.15 a	0.62 ± 0.12 a	0.69 ± 0.28 a	0.58 ± 0.17 a	0.76 ± 0.26 a	0.99 ± 0.65 a	0.33 ± 0.14 a
Arginine	57.14 ± 0.87 a	51.31 ± 8.78 a	15.07 ± 5.25 c	6.54 ± 1.49 cd	41.07 ± 11.69 b	8.45 ± 3.45 cd	4.65 ± 0.74 d	48.06 ± 5.63 ab	6.79 ± 2.27 cd	3.08 ± 1.43 d
Tryptophan	4.18 ± 0.32 ab	0.73 ± 0.08 d	5.43 ± 2.11 a	2.58 ± 0.30 bc	0.49 ± 0.11 d	3.38 ± 1.33 bc	2.41 ± 0.14 c	0.23 ± 0.02 d	3.96 ± 1.50 abc	2.70 ± 0.66 bc
Tyrosine	806.65 ± 44.50 bc	248.34 ± 21.75 c	2541.51 ± 1149.28 a	1105.73 ± 126.02 b	241.29 ± 41.88 c	1507.11 ± 769.14 b	934.06 ± 66.84 bc	236.66 ± 27.35 c	1287.01 ± 544.01 b	834.88 ± 186.75 bc
Histidine	4.29 ± 0.09 c	0.75 ± 1.30 c	18.64 ± 4.78 a	13.15 ± 1.73 b	0.71 ± 1.24 c	15.65 ± 5.45 ab	13.28 ± 2.08 b	1.55 ± 1.35 c	16.46 ± 4.60 ab	11.47 ± 3.08 b
Valine	10.24 ± 0.35 bc	2.96 ± 0.35 c	31.77 ± 16.05 a	12.65 ± 2.03 bc	3.15 ± 0.80 c	19.04 ± 10.02 b	11.09 ± 1.33 bc	3.29 ± 0.58 c	17.26 ± 8.58 b	13.09 ± 4.35 bc
Ornithine	0.00 ± 0.00 e	0.32 ± 0.07 ab	0.21 ± 0.07 bc	0.09 ± 0.08 de	0.41 ± 0.07 a	0.17 ± 0.06 cd	0.09 ± 0.08 de	0.33 ± 0.06 a	0.19 ± 0.07 cd	0.04 ± 0.07 e
Alanine	39.02 ± 0.74 bc	15.19 ± 4.36 c	98.18 ± 38.96 a	69.95 ± 14.23 ab	14.34 ± 3.26 c	66.20 ± 30.13 ab	63.47 ± 11.60 ab	12.16 ± 3.53 c	75.52 ± 31.45 ab	95.63 ± 36.74 a

Taurine	0.00 ± 0.00 a	0.00 ± 0.00 a	0.00 ± 0.00 a	0.00 ± 0.00 a	0.00 ± 0.00 a	0.00 ± 0.00 a	0.00 ± 0.00 a	0.00 ± 0.00 a	0.00 ± 0.00 a	0.00 ± 0.00 a
Isoleucine	8.26 ± 0.57 bc	1.94 ± 0.30 c	34.64 ± 18.09 a	14.64 ± 2.74 b	1.98 ± 0.63 c	19.17 ± 9.86 b	12.01 ± 1.48 bc	1.92 ± 0.25 c	19.44 ± 9.31 b	13.81 ± 4.21 bc
Aspartic acid	3.99 ± 0.21 a	1.21 ± 0.28 bc	0.67 ± 0.34 cd	0.41 ± 0.10 d	0.91 ± 0.48 bcd	0.51 ± 0.27 d	0.61 ± 0.20 d	1.48 ± 0.53 b	0.84 ± 0.38 cd	0.88 ± 0.33 cd
Threonine	1.35 ± 0.06 d	1.37 ± 0.30 d	11.84 ± 4.80 a	6.26 ± 0.86 b	1.38 ± 0.16 d	9.80 ± 4.17 ab	6.37 ± 1.17 b	1.68 ± 0.47 cd	9.15 ± 3.73 ab	5.75 ± 1.53 bc
Glutamine	0.20 ± 0.02 c	1.99 ± 0.40 c	11.29 ± 2.91 b	7.83 ± 1.44 b	2.11 ± 0.38 c	9.21 ± 2.69 b	7.17 ± 1.88 b	2.19 ± 0.62 c	16.10 ± 4.52 a	17.69 ± 5.51 a
Asparagine	14.21 ± 0.51 a	1.18 ± 0.17 d	6.57 ± 2.20 bc	4.59 ± 0.80 c	0.86 ± 0.14 d	5.17 ± 1.82 bc	4.90 ± 1.15 bc	0.90 ± 0.16 d	7.24 ± 2.22 b	6.71 ± 1.98 bc
**Fatty acids (ng/g)**										
Caprylic acid	1337.08 ± 21.16 a	493.81 ± 92.25 b	288.35 ± 14.21 de	297.34 ± 13.71 de	404.06 ± 42.25 c	305.84 ± 6.69 d	313.50 ± 12.18 d	399.29 ± 40.72 c	241.71 ± 21.33 ef	200.44 ± 6.43 f
Decanoic acid	172.50 ± 14.41 a	140.13 ± 23.10 bc	117.24 ± 4.91 cde	111.92 ± 20.86 cde	126.27 ± 21.55 bcd	104.00 ± 23.16 def	98.12 ± 22.18 def	150.58 ± 17.38 ab	92.39 ± 7.55 ef	77.26 ± 10.19 f
Hendecanoic acid	144.46 ± 18.69 a	87.49 ± 21.41 b	64.91 ± 14.83 bc	64.77 ± 4.75 bc	78.85 ± 22.12 bc	74.09 ± 5.99 bc	70.40 ± 0.85 bc	71.64 ± 14.47 bc	66.53 ± 7.71 bc	62.02 ± 4.42 c
Dodecanoic acid	758.67 ± 45.39 a	683.17 ± 19.61 a	405.30 ± 40.16 cd	444.05 ± 57.88 bc	511.69 ± 37.65 b	391.33 ± 35.02 cd	404.83 ± 31.75 cd	471.84 ± 46.54 bc	417.51 ± 97.33 c	330.76 ± 19.23 d
Tridecanoic acid	120.18 ± 16.96 d	450.08 ± 35.63 a	124.23 ± 17.44 d	123.58 ± 7.41 d	350.52 ± 22.24 b	131.86 ± 6.67 d	107.45 ± 12.90 d	231.47 ± 36.53 c	70.43 ± 8.51 e	57.28 ± 6.14 e
Tetradecanoic acid	921.81 ± 107.68 f	6341.10 ± 574.98 a	2602.73 ± 231.61 d	2509.62 ± 194.41 d	5288.89 ± 231.56 b	2586.45 ± 60.48 d	2076.57 ± 131.25 e	4245.90 ± 304.49 c	1195.67 ± 7.26 f	1107.33 ± 54.70 f
Myristoleic acid	17.46 ± 2.65 d	33.79 ± 1.10 a	20.26 ± 2.71 bcd	23.95 ± 2.99 bc	23.59 ± 3.27 bc	18.82 ± 0.81 cd	17.83 ± 3.60 d	21.21 ± 2.68 bcd	24.94 ± 7.33 b	17.71 ± 2.04 d
Myristelaidic acid	36.05 ± 4.27 f	228.40 ± 21.40 a	115.73 ± 10.92 c	86.19 ± 10.29 d	203.62 ± 20.06 b	60.03 ± 6.11 e	53.62 ± 5.68 ef	218.92 ± 14.15 ab	48.98 ± 7.02 ef	53.68 ± 2.86 ef
Pentadecanoic acid	803.77 ± 57.98 f	13172.35 ± 1085.30 a	5557.29 ± 181.63 c	5340.94 ± 298.05 cd	11666.30 ± 1112.48 b	5390.84 ± 533.35 cd	4460.80 ± 574.89 d	11060.01 ± 916.86 b	2462.95 ± 74.24 e	2188.35 ± 317.35 e
cis-10-Pentadecenoic acid	32.10 ± 2.55 e	459.40 ± 31.24 a	92.56 ± 10.69 d	98.03 ± 3.17 d	336.65 ± 71.46 b	93.58 ± 6.60 d	74.57 ± 5.40 de	274.68 ± 38.12 c	35.88 ± 5.81 e	34.32 ± 6.28 e

trans-10-Pentadecenoic acid	16.79 ± 3.89 a	12.74 ± 1.45 a	13.08 ± 1.84 a	12.60 ± 4.88 a	11.35 ± 4.42 a	14.70 ± 2.01 a	13.25 ± 0.57 a	9.94 ± 0.56 a	10.97 ± 3.45 a	10.29 ± 2.32 a
Hexadecanoic acid	21018.79 ± 1536.83 e	189506.92 ± 9948.61 a	106300.23 ± 5524.62 b	102691.36 ± 7869.03 b	178157.64 ± 8096.16 a	107686.14 ± 9027.88 b	88487.31 ± 12895.29 c	179697.39 ± 10181.02 a	52225.19 ± 3036.75 d	42401.47 ± 4980.27 d
Palmitoleic acid	257.10 ± 20.33 f	2236.65 ± 306.20 e	5680.90 ± 86.18 a	5644.28 ± 83.66 a	5379.28 ± 546.72 ab	5054.72 ± 39.83 bc	4275.54 ± 414.34 d	5588.68 ± 208.66 a	4749.20 ± 347.13 cd	4632.26 ± 357.49 cd
Palmitelaidic acid	188.77 ± 50.91 c	555.05 ± 140.01 a	0.00 ± 0.00 d	0.00 ± 0.00 d	439.43 ± 111.12 b	0.00 ± 0.00 d	0.00 ± 0.00 d	441.49 ± 86.45 b	0.00 ± 0.00 d	0.00 ± 0.00 d
Heptadecanoic acid	611.37 ± 57.92 e	8617.01 ± 902.73 a	3881.08 ± 138.55 bc	3635.83 ± 326.61 bc	8133.51 ± 237.59 a	4000.81 ± 506.51 b	3182.56 ± 418.99 c	8130.82 ± 676.03 a	1746.36 ± 233.52 d	1379.26 ± 144.44 d
cis-10-Heptadecenoic acid	291.41 ± 49.66 f	8399.91 ± 526.22 a	3391.42 ± 86.40 c	3055.85 ± 285.84 cd	7084.54 ± 795.84 b	3177.82 ± 263.13 c	2391.99 ± 307.36 d	7314.18 ± 725.90 b	1320.96 ± 56.74 e	1155.60 ± 175.08 e
trans-10-Heptadecenoic acid	47.55 ± 10.39 f	208.44 ± 16.43 a	85.57 ± 11.26 d	78.92 ± 4.83 de	154.63 ± 10.72 c	68.35 ± 3.68 e	64.39 ± 9.73 e	180.81 ± 6.06 b	36.05 ± 4.64 fg	27.77 ± 1.31 g
Octadecanoic acid	9059.65 ± 615.26 d	44731.61 ± 4460.84 a	22054.17 ± 2225.48 c	20767.66 ± 1478.07 c	38082.77 ± 1678.41 b	20528.52 ± 2815.42 c	19172.91 ± 2645.39 c	41097.77 ± 2098.28 ab	11300.22 ± 711.10 d	9573.54 ± 911.15 d
Petroselinic acid	120.79 ± 28.51 de	584.51 ± 81.33 abc	0.00 ± 0.00 e	962.83 ± 214.19 a	830.35 ± 235.78 ab	625.74 ± 658.42 abc	718.76 ± 50.31 ab	950.42 ± 200.82 a	452.39 ± 41.15 bcd	252.43 ± 47.30 cde
Oleic acid	20419.92 ± 933.73 e	157915.96 ± 13061.15 a	92590.02 ± 10897.30 c	88312.46 ± 10984.59 c	122444.99 ± 18516.37 b	76203.27 ± 7725.54 c	74932.60 ± 9191.92 c	118985.22 ± 10739.60 b	42087.63 ± 8433.27 d	29762.93 ± 1956.88 de
cis-Vaccenic acid	19043.05 ± 899.89 e	168054.76 ± 15356.43 a	93342.81 ± 13238.90 c	89656.79 ± 11188.95 c	126141.90 ± 20318.51 b	79219.72 ± 9108.74 c	75025.96 ± 8810.71 c	120808.91 ± 11974.40 b	42175.95 ± 8827.04 d	28668.39 ± 1685.28 de
Petroselaidic acid	58.36 ± 17.71 f	742.01 ± 40.79 b	363.22 ± 41.23 c	302.87 ± 60.36 cd	722.44 ± 199.06 b	256.61 ± 40.74 cde	207.36 ± 23.13 def	937.60 ± 160.74 a	149.74 ± 35.40 ef	108.13 ± 32.72 ef
Elaidic acid	133.96 ± 32.02 e	993.06 ± 159.85 ab	1008.30 ± 53.46 ab	1083.80 ± 292.67 ab	837.00 ± 75.71 bc	1251.59 ± 165.38 a	808.49 ± 312.57 bc	1053.07 ± 177.33 ab	612.85 ± 101.05 cd	381.30 ± 54.74 de
trans-Vaccenic acid	121.65 ± 0.32 f	1124.99 ± 145.92 ab	578.27 ± 34.77 c	586.57 ± 54.06 c	975.20 ± 240.30 b	490.56 ± 54.41 cd	430.26 ± 68.50 cde	1297.59 ± 138.19 a	346.27 ± 10.51 de	274.60 ± 46.73 ef
Linoleic acid	75633.64 ± 4465.21 f	640000.18 ± 17132.37 a	451033.78 ± 14551.50 c	417480.80 ± 41929.18 cd	622020.89 ± 55359.62 a	463621.75 ± 20722.89 c	376516.90 ± 37561.95 d	540303.84 ± 39428.77 b	203945.27 ± 15143.01 e	155014.11 ± 13519.75 e

Linoelaidic acid	142.90 ± 21.15 d	2983.31 ± 338.26 a	1704.99 ± 119.07 b	1680.10 ± 129.86 b	2847.66 ± 449.88 a	1451.19 ± 92.78 bc	1143.45 ± 186.52 c	3239.06 ± 685.90 a	545.87 ± 47.00 d	328.62 ± 54.88 d
trans-7-Nonadecenoic acid	29.46 ± 5.70 g	419.61 ± 15.73 b	141.66 ± 2.66 d	134.34 ± 14.76 d	304.21 ± 10.22 c	109.51 ± 14.35 e	84.17 ± 11.58 f	470.99 ± 29.10 a	75.97 ± 7.98 f	52.24 ± 5.50 g
trans-10-Nonadecenoic acid	18.95 ± 1.73 bc	21.21 ± 1.93 ab	14.02 ± 1.67 d	12.31 ± 1.71 d	20.75 ± 5.16 ab	15.80 ± 1.54 cd	13.68 ± 2.11 d	24.98 ± 4.44 a	13.28 ± 0.99 d	13.17 ± 0.91 d
Arachidic acid	1769.76 ± 88.50 e	32012.79 ± 2851.34 a	13111.66 ± 373.95 c	12914.58 ± 1100.81 c	28597.44 ± 1539.45 b	13406.64 ± 1425.82 c	11208.97 ± 2315.34 c	29359.22 ± 991.25 b	5389.75 ± 1043.09 d	4007.83 ± 490.25 de
gamma-Linolenic acid	640.92 ± 50.92 e	33974.95 ± 4563.50 bc	39946.16 ± 1830.22 a	31880.19 ± 3570.44 bc	37342.50 ± 1313.77 ab	32011.63 ± 566.84 bc	25914.47 ± 3836.71 d	40219.41 ± 4656.75 a	30729.29 ± 3018.65 cd	29247.49 ± 5630.28 cd
cis-11-Eicosenoic acid	39.35 ± 6.04 c	709.54 ± 151.62 a	299.67 ± 25.95 b	290.73 ± 18.67 b	719.39 ± 98.40 a	278.95 ± 15.05 b	252.39 ± 51.32 b	742.25 ± 94.63 a	126.24 ± 7.37 c	84.86 ± 16.21 c
trans-11-Eicosenoic acid	1547.84 ± 72.36 f	39243.32 ± 3939.51 a	18505.49 ± 962.05 c	16839.54 ± 979.20 c	30558.56 ± 2216.32 b	15909.28 ± 1059.05 c	12289.76 ± 1829.01 d	33287.87 ± 3783.21 b	7722.21 ± 915.24 e	5937.68 ± 968.86 e
alpha-Linolenic acid	5798.45 ± 255.69 e	122241.64 ± 5465.05 a	42267.93 ± 4682.88 c	38781.08 ± 2727.71 c	98868.36 ± 9211.10 b	35272.03 ± 1600.84 c	25403.84 ± 3575.71 d	90862.32 ± 8415.99 b	9714.23 ± 569.29 e	6308.78 ± 637.07 e
Heneicosanoic acid	382.44 ± 28.63 d	10031.84 ± 1273.10 a	3791.13 ± 190.36 c	3495.44 ± 289.38 c	8684.76 ± 579.92 b	4050.50 ± 810.61 c	3078.72 ± 529.79 c	9396.37 ± 1468.07 ab	1384.54 ± 242.91 d	1089.20 ± 138.40 d
cis-11,14-Eicosadienoic acid	356.04 ± 23.95 f	10887.45 ± 1033.12 a	5726.36 ± 298.00 c	5331.39 ± 506.88 cd	9336.69 ± 763.30 b	5151.11 ± 163.04 cd	4435.56 ± 721.84 d	9997.17 ± 576.97 ab	2743.16 ± 167.93 e	2347.40 ± 510.06 e
Docosanoic acid	1302.61 ± 107.88 d	29008.74 ± 2271.25 a	12997.93 ± 593.21 b	11781.97 ± 1327.99 b	28847.94 ± 3219.47 a	13132.95 ± 1484.15 b	10412.22 ± 2269.88 b	29795.76 ± 916.28 a	4986.52 ± 788.31 c	4168.34 ± 337.32 c
homo-gamma-Linolenic acid	46.87 ± 4.10 f	732.49 ± 55.45 a	479.89 ± 37.68 c	448.90 ± 28.88 c	623.17 ± 79.12 b	370.55 ± 20.70 d	339.97 ± 44.27 de	759.13 ± 28.34 a	275.57 ± 40.84 e	311.86 ± 38.34 de
Erucic acid	0.00 ± 0.00 e	58.17 ± 9.23 a	23.35 ± 1.97 c	23.14 ± 1.77 c	46.76 ± 7.83 b	22.56 ± 2.98 c	20.22 ± 1.70 cd	57.99 ± 7.05 a	14.20 ± 2.75 d	12.92 ± 2.30 d
Brassidic acid	63.48 ± 3.53 e	1366.07 ± 148.00 a	719.19 ± 65.25 c	722.51 ± 150.54 c	955.12 ± 72.04 b	536.68 ± 61.66 d	531.20 ± 46.42 d	1471.95 ± 155.50 a	495.21 ± 66.83 d	494.41 ± 72.02 d
cis-11,14,17-Eicosatrienoic acid	40.71 ± 1.15 g	705.72 ± 36.40 a	421.65 ± 5.68 c	430.06 ± 21.40 c	572.55 ± 76.01 b	360.21 ± 13.85 cd	330.91 ± 49.68 de	683.88 ± 64.28 a	250.01 ± 22.56 f	278.79 ± 49.47 ef
Arachidonic acid	60.78 ± 5.97 a	46.44 ± 9.87 b	35.03 ± 4.40 c	42.70 ± 5.55 bc	49.32 ± 9.15 b	42.81 ± 3.35 bc	48.11 ± 2.92 b	42.42 ± 5.65 bc	34.19 ± 1.70 c	36.30 ± 3.62 c
Tricosanoic acid	1410.64 ± 187.86 d	24512.60 ± 3087.72 b	9109.50 ± 847.04 c	8384.12 ± 520.47 c	22412.84 ± 1638.90 b	9565.82 ± 1394.63 c	8592.96 ± 2569.39 c	27234.43 ± 1463.59 a	3279.44 ± 645.56 d	2636.94 ± 593.08 d

cis-13,16-Docosadienoic acid	174.59 ± 13.83 f	5161.71 ± 201.22 ab	2395.90 ± 50.94 c	2227.00 ± 304.95 cd	4655.85 ± 537.87 b	2238.90 ± 356.61 cd	1722.05 ± 331.97 d	5382.36 ± 560.08 a	945.51 ± 123.54 e	784.29 ± 79.34 e
cis-5,8,11,14,17-Eicosapentaenoic acid	208.50 ± 52.23 a	86.86 ± 8.07 b	95.86 ± 30.63 b	110.72 ± 20.05 b	109.05 ± 18.55 b	127.18 ± 38.88 b	102.98 ± 9.76 b	96.93 ± 16.99 b	94.26 ± 16.81 b	99.41 ± 26.13 b
cis-7,10,13,16-Docosic acidtraenoic acid	14.70 ± 13.00 a	0.00 ± 0.00 a	3.70 ± 6.41 a	3.76 ± 6.51 a	0.00 ± 0.00 a	8.91 ± 7.96 a	0.00 ± 0.00 a	3.39 ± 5.87 a	0.00 ± 0.00 a	0.00 ± 0.00 a
cis-7,10,13,16,19-Docosapentaenoic acid	32.56 ± 2.18 a	12.20 ± 2.03 bc	13.71 ± 2.14 bc	15.84 ± 4.66 bc	9.96 ± 9.83 c	16.63 ± 1.99 bc	14.78 ± 1.61 bc	12.34 ± 1.12 bc	14.33 ± 1.60 bc	17.03 ± 3.75 b
cis-4,7,10,13,16-Docosapentaenoic acid	17.97 ± 3.44 a	5.51 ± 0.28 d	7.44 ± 1.23 cd	6.82 ± 2.30 cd	9.65 ± 3.52 bc	9.30 ± 1.20 bc	12.65 ± 1.59 b	8.60 ± 1.60 cd	9.52 ± 1.44 bc	10.40 ± 2.57 bc
Tetracosanoic acid	1989.16 ± 210.50 e	21700.86 ± 620.41 b	12062.44 ± 945.89 c	13097.05 ± 71.49 c	21445.06 ± 926.35 b	12619.09 ± 1488.78 c	13483.67 ± 2520.04 c	25180.83 ± 1996.07 a	7379.87 ± 449.13 d	8017.54 ± 911.60 d
Nervonic acid	60.73 ± 7.74 e	1155.14 ± 128.41 a	472.56 ± 13.52 c	461.66 ± 66.55 c	712.28 ± 76.55 b	281.58 ± 39.86 d	277.69 ± 34.79 d	1163.19 ± 102.72 a	288.40 ± 20.38 d	281.11 ± 31.43 d
cis-4,7,10,13,16,19-Docosahexaenoic acid	153.37 ± 25.69 d	1045.94 ± 172.50 ab	350.55 ± 202.40 cd	385.70 ± 12.76 cd	908.51 ± 139.06 b	492.34 ± 38.67 c	310.96 ± 84.75 cd	1274.20 ± 360.24 a	259.52 ± 85.77 cd	180.76 ± 33.69 d

Note: Means for individual compounds were compared using one-way ANOVA followed by Fisher's LSD post hoc test. Values are presented as mean ± standard deviation. Different lowercase letters indicate significant differences ($p < 0.05$). Concentrations are expressed on a fresh weight basis.
